# MicroRNA-mediated regulation of lipid metabolism in virus-infected *Emiliania huxleyi*

**DOI:** 10.1038/s41396-022-01291-y

**Published:** 2022-07-22

**Authors:** Enquan Zhang, Jingjing Gao, Zehua Wei, Jun Zeng, Jian Li, Guiling Li, Jingwen Liu

**Affiliations:** College of Ocean Food and Biological Engineering, Jimei University, Xiamen, 361021 China

**Keywords:** Small RNAs, Metabolomics, Transcriptomics, Virus-host interactions, Next-generation sequencing

## Abstract

The interactions between *Emiliania huxleyi* and *E. huxleyi* virus (EhV) regulate marine carbon and sulfur biogeochemical cycles and play a prominent role in global climate change. As a large DNA virus, EhV has developed a novel “virocell metabolism” model to meet its high metabolic needs. Although it has been widely demonstrated that EhV infection can profoundly rewire lipid metabolism, the epigenetic regulatory mechanisms of lipid metabolism are still obscure. MicroRNAs (miRNAs) can regulate biological pathways by targeting hub genes in the metabolic processes. In this study, the transcriptome, lipidome, and miRNAome were applied to investigate the epigenetic regulation of lipid metabolism in *E. huxleyi* cells during a detailed time course of viral infection. Combined transcriptomic, lipidomic, and physiological experiments revealed reprogrammed lipid metabolism, along with mitochondrial dysfunction and calcium influx through the cell membrane. A total of 69 host miRNAs (including 1 known miRNA) and 7 viral miRNAs were identified, 27 of which were differentially expressed. Bioinformatic prediction revealed that miRNAs involved in the regulation of lipid metabolism and a dual-luciferase reporter assay suggested that phosphatidylinositol 3-kinase (PI3K) gene might be a target of ehx-miR5. Further qPCR and western blot analysis showed a significant negative correlation between the expression of ehx-miR5 and its target gene *PI3K*, along with the lower activity of its downstream components (p-Akt, p-TOR, SREBP), indicating that lipid metabolism might be regulated by ehx-miR5 through the PI3K-Akt-TOR signaling pathway. Our findings reveal several novel mechanisms of viral strategies to manipulate host lipid metabolism and provide evidence that ehx-miR5 negatively modulates the expression of PI3K and disturbs lipid metabolism in the interactions between *E. huxleyi* and EhV.

## Introduction

The coccolithophore *Emiliania huxleyi* frequently forms large oceanic algal blooms in subpolar waters, which are significant contributors to the global production and export of calcium carbonate (calcite) [1–3]. The collapse of massive *E. huxleyi* blooms is frequently terminated by the infection of a specific large double-stranded DNA (dsDNA) virus (*E. huxleyi* virus, EhV) [4, 5]. *E. huxleyi*–EhV interactions therefore play a profound role in determining carbon flow in the ocean and global climate processes [6–8]. As a relatively large dsDNA virus, EhV has evolved complex and dynamic interactions with its host cells [9] by which it can reshape its host’s metabolic network, generating the virocell metabolic state that supports its specific requirement [10]. For example, EhV depends on host metabolic networks to supply essential building blocks such as amino acids, nucleotides, and fatty acids (FAs) for the production of new virions [6, 11, 12]. Recent studies demonstrated that, in addition to being passive consumers of host metabolic products, EhVs could expand the metabolic capabilities of their hosts by introducing virus-encoded auxiliary metabolic genes (vAMGs) to support their specific requirements [10]. In particular, virus-encoded enzymes induce a metabolic switch in sphingolipid metabolism to synthesize unique virus-specific glycosphingolipids, which are required for virus assembly and egress [13, 14]. The realization of these multiple metabolic strategies in the virocells may involve the regulation of gene expression at multiple levels, ranging from transcriptional, posttranscriptional, translational, to posttranslational. The regulation of transcriptional level is the principal regulating means of gene expression; however, posttranscriptional (such as via microRNAs (miRNAs)) regulation plays an important role in the process of gene expression.

miRNAs are small noncoding RNAs that are involved in posttranscriptional regulation of their target genes in a sequence-specific manner [15]. As one of the most abundant classes of gene regulators, miRNAs are involved in many biological processes, such as cell proliferation, tumorigenesis, apoptosis, and inflammation [16, 17]. Furthermore, miRNAs control plant development, growth, stress adaptation, and other physiological processes [18, 19]. Recent studies have indicated that miRNAs also play novel roles in regulating metabolic pathways through the control of target genes involved in metabolic processes, and especially in controlling lipid and glucose metabolism [20, 21]. Emerging evidence demonstrates that miRNAs are critical regulators of lipid synthesis, FA oxidation, and lipoprotein formation and secretion [22–25]. Dysregulation of miRNAs disrupts the gene regulatory network, leading to metabolic syndrome and its related diseases in human beings [26]. To date, more than 30 miRNAs in mammalian cells have been identified to play crucial roles in the regulation of lipid metabolism through various pathways [21], such as FA oxidation, FA biosynthesis, cholesterol, and triacylglycerol [17, 25, 27]. In marine phytoplankton, miRNAs are involved in regulating phosphorus utilization in the dinoflagellate *Prorocentrum donghaiense* in response to phosphorus stress [28]. Moreover, both in higher animals and plants, miRNAs have been shown to play an important role in the virus-host arms race, including cellular antiviral responses and/or in the promotion of viral infection through complex regulatory pathways [29, 30]. This regulatory network represents a new and central role for miRNAs in lipid and antiviral regulation. Besides, it has been confirmed that viruses can also encode miRNAs. miRNAs are particularly useful tools for DNA viruses since they are nonimmunogenic for host cells, and thus miRNAs can potentially manipulate host cells without evoking host immune responses [30]. Zhang et al. [31] firstly reported the existence of miRNAs in the coccolithophore *E. huxleyi*, indicating that this form of posttranscriptional regulation might be a major mechanism controlling gene expression. Indeed, it has been found that miRNAs can be produced in the form of extracellular vesicles during EhV infection, which might serve as signal molecules to target sphingolipid metabolism and cell-cycle pathways [32]. To date, the regulatory roles of both host and viral miRNAs in metabolic pathways (especially in lipid metabolism) in the *E. huxleyi*–EhV system are largely unexplored.

In this study, we combined lipidome, transcriptome, and miRNAome analysis in *E. huxleyi* (BOF92) and its specific lytic virus (EhV99B1), a marine model host–virus system. Our data provide the most comprehensive insights to date into how EhVs employ diverse strategies to manipulate host lipid metabolism with some novel mechanisms while demonstrating a potential miRNA regulatory mechanism of lipid metabolism in the *E. huxleyi*–EhV system.

## Materials and methods

### Algal culture and viral infection

The *E. huxleyi* strain BOF92 and EhV (EhV99B1) used in this study were isolated from the west coast of Norway (60°24′N, 5°19′E). Cultures of *E. huxleyi* BOF92 were grown in f/2-Si medium at 16 °C with a 14:10 h light/dark illumination cycle. A light intensity of ~100 μM photons·m^−2^·s^−1^ was provided by cool white fluorescent lights. Exponentially growing cells (~7.5 × 10^5^ cell·mL^−1^) were infected with a 1:50 volumetric ratio of virus lysate to culture (multiplicity of infection of ~1:1 viral particle per cell).

### Sample collection

At ca. 0–96 h post inoculation (hpi), samples (2 mL) for algal and viral counts were fixed with 1.0% and 0.5% glutaraldehyde (final concentration) respectively, frozen in liquid nitrogen, and stored at −80 °C until analysis. At ca. 0, 6, 12, 24, 48, and 60 hpi, i.e., the early, middle, and late stages in the second lytic cycle when the majority of the cells were infected, 100 mL of the cultures was harvested by centrifugation at 3000 rpm for 10 min at 4 °C, and the cell pellets were fixed (4% formaldehyde + 1% glutaraldehyde in 0.1 M phosphate buffer, pH 7.4) for 4 h at 4 °C for sample preparation for transmission electron microscopy (TEM). The cell pellets of 1000 mL of cultures at the time points indicated were collected for RNA isolation, protein extraction, biochemical experiments (three biological replicates), and total lipids isolation (six biological replicates).

### RNA isolation, library construction, and sequencing

Total RNA was extracted using TRIzol Reagent kit (Invitrogen, Carlsbad, CA, USA) according to the manufacturer’s instructions. RNA quality was assessed on an Agilent 2100 Bioanalyzer (Agilent Technologies, Palo Alto, CA, USA) and the RNA integrity numbers of the samples were all above 6.0. Then, these isolated RNAs were used for sequencing and quantitative PCR analysis. Equal amounts of RNA masses extracted from the triplicate samples were pooled for RNA sequencing.

A total of 3 μg of RNA was used to generate paired-end RNA-seq library using NEBNext Ultra II Directional RNA Second Strand Synthesis Module (NEB, Ipswich, MA, USA). Small RNA libraries were generated using NEBNext Multiplex Small RNA Library Prep Set for Illumina (NEB, Ipswich, MA, USA) with the same amount of total RNA mass as used in RNA-seq. The libraries were purified (AMPure XP system), quantified using the Agilent Bioanalyzer 2100 system, and then sequenced using HiSeq 4000 and HiSeq 2500 for mRNA and small RNA sequencing, respectively. The raw data were deposited to NCBI in SRA (BioProject accession number: PRJNA705400). The Transcriptome Shotgun Assembly project has been deposited at DDBJ/EMBL/GenBank under the accession GJZP00000000. The version described in this paper is the first version, GJZP01000000. The library preparation and deep sequencing were performed by Genedenovo Biotechnology Co., Ltd (Guangzhou, China).

### Bioinformatics analysis

Raw reads for RNA-seq and miRNA sequencing were cleaned up by trimming adaptor sequences, removing poly-N-containing reads, and filtering low-quality reads (*Q* score ≤20 for ≥50% of nucleotides in each read) using fastp (version 0.18.0) [33]. All downstream analyses were based on clean data with high quality.

Host transcriptome de novo assembly was carried out using the short read assembly program Trinity [34], with the two sets of sequence reads as the input. Basic annotation of unigenes included protein functional annotation, pathway annotation, KOG functional annotation, and Gene Ontology (GO) annotation using the BLASTx program with an *E*-value threshold of 1e–5. Protein functional annotations could then be obtained according to the best alignment results. The gene abundances were calculated and normalized to reads per kilobase per million mapped reads (RPKM) [35]. For the assembly of viral transcriptome, an index of the reference genome was built, and paired-end clean reads were mapped to the reference genome (https://www.ncbi.nlm.nih.gov/nuccore/FN429076.1) using HISAT v2.2.4 [36] with “-rna-strandness RF” and other parameters set as a default. The mapped reads of each sample were assembled by using StringTie v1.3.1 [37, 38] in a reference-based approach. For each transcription region, a fragment per kilobase of transcript per million mapped reads value was calculated to quantify its expression abundance and variations. RNA differential expression analysis was performed by DESeq2 software [39] between two different groups. Genes with a false discovery rate below 0.05 and absolute fold change (FC) ≥2 were considered differentially expressed (DE) genes.

For the miRNA sequencing dataset, reads less than 18 nt in length were removed. All clean tags were aligned with small RNAs in the GenBank database [40] (Release 209.0) and Rfam database [41] (Release 11.0) to identify and remove rRNA, scRNA, snoRNA, snRNA, and tRNA. To avoid omitting the miRNAs located in intergenic regions or introns, all clean tags were aligned with both the genome of *E. huxleyi* CCMP1516 (https://www.ncbi.nlm.nih.gov/assembly/GCF_000372725.1) and the de novo transcriptome. The tags mapped to repeat sequences were removed. All clean tags were then searched against the miRBase database [42] (Release 22) to identify existing miRNAs. To date, the miRNA sequences of some species of phytoplankton have not been included in the miRBase database. The miRNA alignment with other species was a dependable way to identify the known miRNAs. According to their miRNA positions and hairpin structures predicted by Mireap_v0.2 software [43], novel miRNA candidates were identified. The default parameters of Mireap_v0.2 software were as follows: miRNA sequence length, 18–25 nt; miRNA reference sequence length, 20–23 nt; maximal copy number of miRNAs on reference, 20; maximal free energy allowed for a miRNA precursor, 18 kcal/mol; space between miRNA and miRNA*, 16–300 nt; maximal bulge between miRNA and miRNA*, 4 nt; maximal asymmetry of miRNA/miRNA* duplex, 4 nt; flank sequence length of miRNA precursor, 20 nt. The miRNA expression level was calculated and normalized to transcripts per million. After normalization, the miRNA expression profiles between the two miRNA libraries were compared. Differential expression analysis of two conditions was performed using the DESeq2. DE miRNAs were defined as those with an FC ≥2 and *p* value <0.05. Based on the sequences of the known miRNAs and novel miRNAs, the CDS and 3′ UTR sequence of each unigene transcript were used to predict both animal- and plant-type targets. miRanda [44] (miRanda-3.3a, -sc 140 -en -15 -strict) was used to predict targets with animal miRNA binding characteristics, while plant-type targets were predicted by patmatch software [45] (Version 1.2) with default parameters. The targets with Pearson correlation >−0.3 with their miRNAs were not included in the subsequent analysis.

Based on the high correlation of genes, gene modules were divided by weighted gene coexpression network analysis (WGCNA) [46], a systems biology method for describing the correlation patterns among genes across multiple samples. The DE unigenes expressing in more than half (nine) samples and with relatively high expression levels (the sum of RPKM >18) were retained, getting 26,473 genes for subsequent analysis. After filtering genes, gene expression values were imported into WGCNA, an R package [46], to construct coexpression modules using the automatic network construction function blockwiseModules with default settings, except that the power was 16 and minModuleSize was 100.

Pathway enrichment analysis identified significantly enriched metabolic pathways or signal transduction pathways in the analyzed genes compared with the whole genome background. All the module genes and miRNA target genes were considered in our analysis. The formula used to calculate the *p* value is based on the hypergeometric distribution test. The calculated *p* value was corrected by *Q* value, taking *Q* ≤ 0.05 as a threshold. Pathways meeting this condition were defined as significantly enriched pathways.

### Lipidomics analysis

Nontargeted lipidomics analysis was performed with an ACQUITY ultra-performance liquid chromatograph (Waters, USA) coupled with a Q-Exactive HF mass spectrometer (Thermo Fisher Scientific, USA). The methods for sample preparation, equipment, and mass spectrometry (MS) analysis were based on our previously published article [12]. The lipid internal standards utilized in this study are provided in the Supplementary information.

For data processing, the identification of lipid species was performed based on accurate m/z, tandem mass spectrometry (MS/MS) fragmentation patterns, and retention behavior using the LIPID MAPS database, and LipidSearch and Xcalibur software (Thermo, USA). Then, the lipids were quantified at an m/z tolerance of ±5 ppm and a retention time extraction window of ±15 s using Trace Finder software (Thermo, USA), and normalized to the peak areas of internal standards. Quality evaluation of lipid profiling and multivariate analysis with unit variance scaling were performed by Simca (v14.1) software (Umetrics, Sweden). Heatmaps for lipid profiles were generated and visualized by the R program. Lipid metabolic pathways were analyzed with reference to the LIPID MAPS and Kyoto Encyclopedia of Genes and Genomes (KEGG) databases. The lipidomics data have been deposited to the EMBL-EBI MetaboLights database [47] with the identifier MTBLS5178. Abbreviations for lipid classes used in this study are as follows: FA, fatty acid; OAHFA, (O-acyl)−1-hydroxy fatty acid; MG, monoglyceride; MGDG, monogalatosyl diglyceride; DG, diglyceride; TG, triacylglycerol; PC, phosphatidylcholine; PE, phosphatidylethanolamine; PG, phosphatidylglycerol; PA, phosphatidic acid; PI, phosphatidylinositol; PS, phosphatidylserine; CL, cardiolipin; Cer, ceramide; SM, sphingomyelin; GSL, glycosphingolipid; WE, wax ester; and CmE, cholesterol.

### Quantitative real-time PCR

Reverse transcription quantitative PCR (qRT-PCR) was performed to determine the transcriptional levels of several genes involved in lipid metabolism. The primers used for qPCR are listed in Supplementary Table S1 (mRNAs) and Supplementary Table S2 (miRNAs). For miRNA qRT-PCR, a miRcute Plus miRNA First-Strand cDNA Kit (TIANGEN, Beijing, China) was used to facilitate cDNA synthesis. Then, the RT product was used as the template for qPCR. All qPCRs were performed on a Roche LightCycler 480II/96 Real-time PCR System (Roche, Switzerland) using Universal SYBR Green Supermix (Vazyme, Nanjing, China) in 96-well plates according to the manufacturer’s recommendations. U6 snRNA and CDKA [48] were used as references to calibrate the expression of the miRNAs and mRNA, respectively. Two technical and three biological replicates were conducted for both viral infection and control culture samples. The comparative threshold (2^−ΔΔCt^) [49] method was used to assess the relative expression levels.

### Dual-luciferase reporter assay

Dual-luciferase reporter assays were performed using recombinant vectors carrying either the wild-type or mutated sequence of the phosphatidylinositol 3-kinase (PI3K) gene cotransfected into HepG2 cells with miRNA or NC mimics. The complete CDS of *PI3K* containing the putative binding sites of ehx-miR5 was cloned into the psi-CHECK2 vector to obtain the wild-type vectors. Mutated vectors were obtained via the method by which mutations were prepared in the seed sequences where ehx-miR5 was predicted to be completely complementary with it on the wild-type vector. All constructs were verified by DNA sequencing. For the transfection experiments, HepG2 cells were cultured in 24-well plates with 500 μL of medium and grown to approximately 70% confluence. HepG2 cells were cotransfected with 600 µg recombinant vectors and 20 pmol of miRNA mimics (or NC mimics) by Lipofectamine 2000 (Invitrogen, Carlsbad, CA, USA). Cells transfected with recombinant vectors alone were employed as blank group. After 48 h of transfection, the cells were prepared for detecting relative luciferase activity following the protocols of the Dual-Luciferase Reporter Assay Kit (Vazyme, Nanjing, China). Each trial contained three replicates, and the experiments were repeated twice.

### Western blotting

A standard western blotting protocol was used as described previously [50]. Briefly, control and virus-infected cells were lysed in cell lysis buffer. The concentration of total protein was determined using a BCA Protein Assay Reagent kit (SuperSignal West Dura Trial kit, Pierce, Covance, USA). Equal amounts (40–60 μg) of protein were electrophoresed, transferred to nitrocellulose membranes, and incubated with the primary antibody. Peroxidase-conjugated secondary antibody and a WesternBright ECL western blotting detection kit (Advansta, Menlo Park, CA, USA) were used to detect the signals with the Western Blotting System and analyzed with the Azure c400 Imaging System (Azure Biosystems, USA). In addition, subcellular fractionation experiments were used to estimate the expression of the molecules in the nuclear fraction including lipin 1 and sterol regulatory element-binding protein (SREBP). Nucleoproteins were separated from cells using the nuclear and cytoplasmic protein extraction kit (Beyotime, Shanghai, China) (specific procedures are shown in the Supplementary information). CDKA and histone 3 (H3) were used as the loading controls. The primary antibodies used are shown in Supplementary Table S3.

### Statistical analysis

The results are displayed as the mean and standard deviation from at least three independent experiments. Statistical analysis was performed by Student’s *t* test (for two groups) or analysis of variance (for more than two groups) followed by SNK’s or Duncan’s tests, and a *p* value or corrected *p* value less than 0.05 was considered statistically significant. All analyses were conducted in SPSS 25.0.

## Results and discussion

### Infection dynamics of *E. huxleyi* and neutral lipid accumulation

**Fig. 1:**
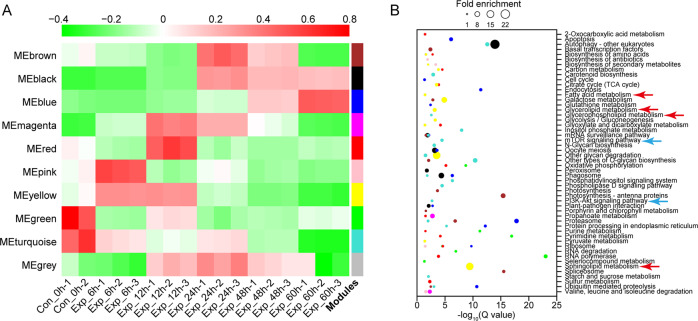
Global transcriptomic profiles and pathway enrichment analysis during the large-scale infection process. **A** Global gene expression profiles of host genes during infection. Different modules were generated by WGCNA and distinguished with different colors. **B** Significantly enriched KEGG pathways (*Q* < 0.05) related to host gene clusters as displayed in (**A**). Colors refer to clusters as indicated in (**A**).

We exposed *E. huxleyi* BOF92 to its lytic virus EhV99B1 and followed its dynamics over the time course of infection. Cultures infected with the viruses showed growth arrest and subsequent lysis (Supplementary Fig. S1A), along with a loss of brown pigmentation with the extension of infection time (Supplementary Fig. S1B–G). TEM revealed that lipid bodies accumulated in the cytoplasm and around the cell membrane at 24 hpi compared with 0 hpi (Supplementary Fig. S1H–M). At 48 hpi, the observation of viral particles within intracytoplasmic vacuoles and the assembled virions indicated the development of viral infection (Supplementary Fig. S1K, L). Prolific virus release could be observed at 72 hpi (Fig. 1M). Moreover, fluorescence microscopy imaging showed a strong punctuated BODIPY 493/505-stained fluorescence signal in cells infected with lytic viruses that was not observed in uninfected cells (Supplementary Fig. S1N), suggesting the accumulation of neutral lipids in lipid droplets during lytic infection.

### Virus-driven lipid metabolism reprogramming

De novo transcriptome was conducted to identify critical genes involved in lipid metabolism during viral infection. The sequencing statistics (Supplementary Table S4), assembly quality statistics (Supplementary Table S5), and annotation information (Supplementary Table S6 and Supplementary Fig. S2) were provided in Supplementary information. Principal component and sample correlation analysis showed that one sample (Con_0h-3) was an extreme outlier (Supplementary Fig. S3), which was not included in the following analysis. We cannot rule out the possibility of potential contamination introduced during RNA-seq library construction. A total of 26,473 unigenes were clustered into nine co-expressed modules (MEs) (genes not belonging to any other ME were clustered into MEgery) by WGCNA (Supplementary Fig. S4), and MEs with different expression trends were distinguished by different colors (Fig. 1A). KEGG enrichment analysis of the unigenes in different MEs (Fig. 1B) showed that FA metabolism (MEyellow), sphingolipid metabolism (MEyellow), glycerolipid metabolism (MEyellow), and glycerophospholipid metabolism (MEturquoise) were significantly altered with the duration of infection (Fig. 1B, noted by red arrows), which were supported by our nontargeted lipidomic analysis results (Supplementary Figs. S5–S9, Supplementary Table S7, and Supplementary Dataset 1). Moreover, the PI3K-Akt and mTOR signaling pathways, one of the most important pathways participating in the regulation of glucose and lipid metabolism in mammalian cells [51] were enriched in MEturquoise (Fig. 1B, noted by blue arrows). In addition, a relaxed cut-off (FC ≥1.5) was also used to determine DE genes, and 30,048 DE genes were screened, which enriched several more pathways besides those that existed based on FC ≥2.0 (data not shown here). The analysis of the viral gene expression profiles  revealed that almost all genes were explosively upregulated at 48 hpi (Supplementary Fig. S10), including the vAMGs participating in sphingolipid and glycerolipid metabolism. Thirty-one genes (including six viral genes) (Supplementary Table S8) participating in lipid metabolism were validated by qRT-PCR (Supplementary Fig. S11). Integrated transcriptomic and lipidomic pathway analysis (Supplementary Fig. S12–S14 and Supplementary Dataset 2) revealed TG accumulation and a metabolic shift toward viral sphingolipid metabolism (more details are provided in Supplementary results), which were consistent with those of the previous reports [11–13].

### Cardiolipins and calcium-mediated lipid accumulation in EhV-infected cells

The sum of CLs in our lipidomic data showed a significant decrease at the late stages of virus infection (48–60 hpi), whereas we noticed a significant accumulation of CLs at 24 hpi, accompanied by increased abundance in PGs, the precursor of CL synthesis and the CL synthase gene (CRLS) levels from 6 to 24 hpi (Supplementary Fig. S13). CL is the signature lipid of the mitochondrial inner membrane and any change in CL may cause alterations in mitochondrial function [52]. Several important mitochondrial indexes were detected and the results revealed gradually increased cellular reactive oxygen species levels (Supplementary Fig. S15A), reduced mitochondrial membrane potential (Supplementary Fig. S15B), and ATP levels (Supplementary Fig. S15C) during EhV infection. Compared with that at 0 and 6 hpi, the ATP concentrations barely changed at 24 hpi, although it declined significantly at 48 hpi (Supplementary Fig. S15C), suggesting that CLs alleviated the impaired mitochondrial electron transport function to transiently maintain the essential ATP level.

In addition, cellular calcium influx is important for transcriptional programming of lipid metabolism, including lipid storage and lipolysis [53–55]. To test whether the influx of calcium is required for cellular lipogenesis and lipid storage by controlling calcium homeostasis, we detected the Ca^2+^ influx across the cell membrane at 0–24 hpi by the Non-invasive Micro-test Technology (Supplementary materials and methods). The results showed a significant influx of extracellular Ca^2+^ at 12 and 24 h after viral infection (Supplementary Fig. S15D). We speculated that exogenous Ca^2+^ influx might lead to an increase in mitochondrial Ca^2+^ level and maintenance of mitochondrial calcium homeostasis and TCA cycle metabolites, hence providing indispensable energy for lipogenesis, CL remodeling, and lipid droplet storage during EhV infection. Altogether, our findings suggested that cellular calcium ions might be important for maintaining lipid homeostasis in the middle phase (24 hpi) of virus-infected *E. huxleyi* cells.

### Identification and quantification of host and viral miRNAs

**Fig. 2:**
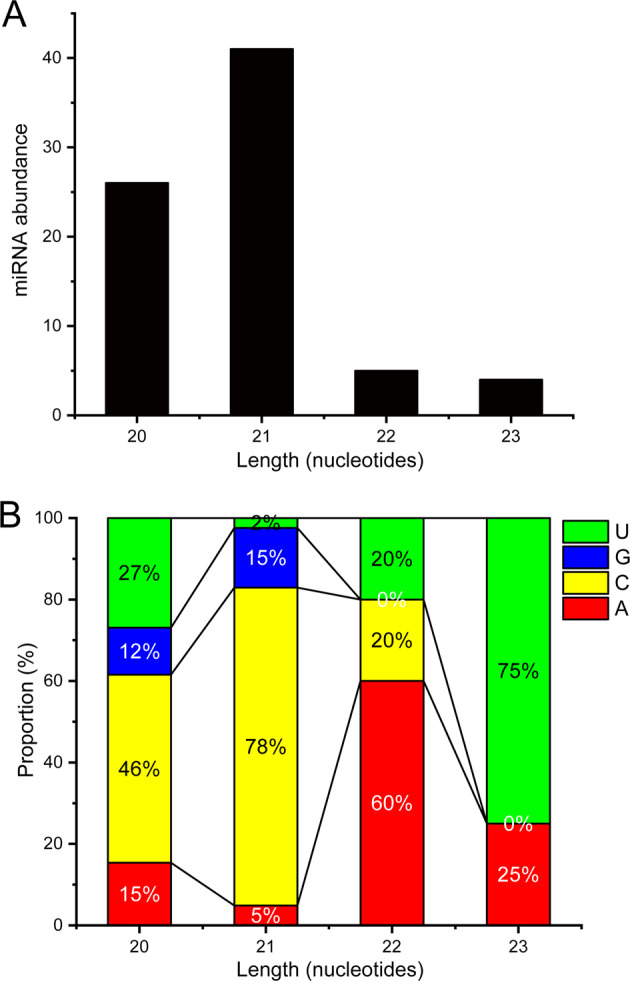
The basic characters of miRNAs identified in virus-infected *E. huxleyi*. **A** Length and number distribution of miRNAs. **B** First nucleotide bias of miRNAs.

For miRNA identification, given that miRNAs might be derived from coding regions or noncoding regions, the genome of *E. huxleyi* CCMP1516 and our de novo transcriptome were both considered to be the mapping reference. Approximately 57.45% of the small clean tags were left after the filtration to clean reads (Supplementary Dataset 3). As a result, 69 host mature miRNAs and 7 viral miRNAs were identified, 1 host being known and 68 being novel miRNAs (the details are shown in Supplementary Dataset 4). The lengths of these miRNAs ranged from 20 to 23 nt with a peak at 21 nt (Fig. 2A), which agreed with typical miRNAs in plants with length peaks at 21 nt. The first nucleotide of these miRNAs had a bias for C (Fig. 2B), consistent with the first nucleotide bias of miRNAs identified in *E. huxleyi* CCMP1516 [31]. Twenty host mature miRNAs were differentially expressed in at least one comparative group and because all viral miRNAs were only expressed at 48 and 60 hpi, these seven miRNAs were all considered DE miRNAs (Supplementary Dataset 5). These DE miRNAs were named according to general miRNA nomenclature and the known miRNA (miR-574-5p) was named ehx-miR5 (Supplementary Dataset 5, the hairpin structures of DE miRNAs are shown in Supplementary Fig. S16). All DE miRNAs were verified by qRT-PCR (Supplementary Table S9), and the qPCR results were subject to regression analysis (*R* = 0.87, Supplementary Fig. S17).

### Bioinformatic prediction of miRNA functions reveals that miRNAs might mediate the PI3K-AKT signaling pathway

Plant- and animal-type target genes were both analyzed in this study. Based on plant-like binding characteristics, 382 unigenes were targeted by host DE miRNAs, while virus-derived miRNAs only targeted three unigenes (unigene0025568, transketolase 1; unigene0047618, phenylalanine--tRNA ligase beta subunit; unigene0055743, N-acetylglutamate synthase). The 382 plant-type target genes were subjected to GO and KEGG functional analysis. Most GO terms did not seem to be related to lipid metabolism (Supplementary Fig. S18A). In KEGG annotation (Supplementary Fig. S18B), eight unigenes possibly participated in lipid metabolism (Supplementary Table S10), but the pathways to which these unigenes belonged were not significantly enriched. To date, the miRNA binding characteristics in *E. huxleyi* are largely unknown, although the existence of miRNAs has been confirmed [31]. Nevertheless, a previous study demonstrated that target hybridization to nucleotides of the seed region was sufficient to induce moderate repression of expression in another species of unicellular algae, *Chlamydomonas reinhardtii*, which might imply that the base-pairing requirements for small RNA-mediated repression were more similar to those of metazoans in *C. reinhardtii* [56]. Therefore, miRNA function analysis was subsequently focused on animal-type target genes.

**Fig. 3:**
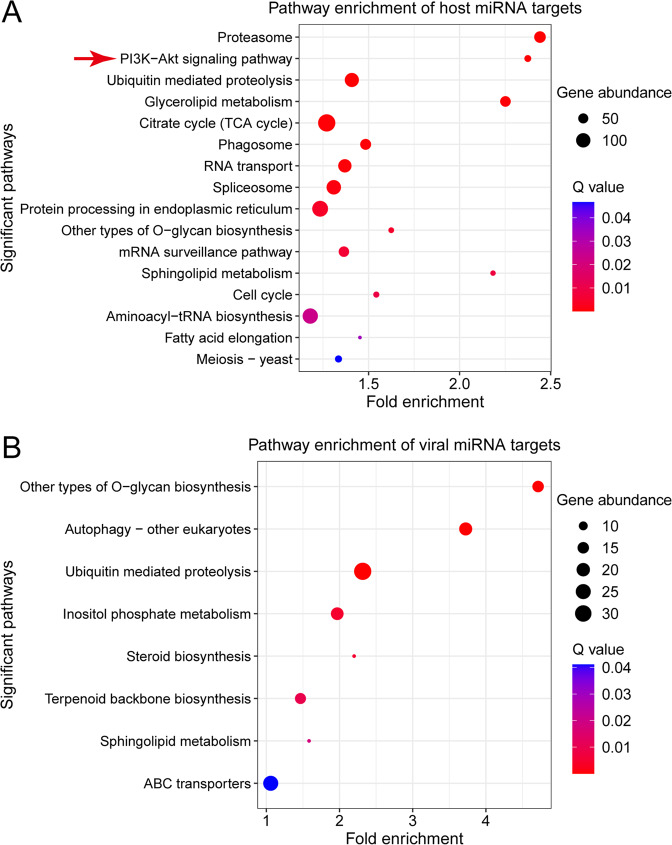
KEGG enrichment analysis of miRNA animal-type targets. **A** Enrichment analysis of host DE miRNA targets. **B** Enrichment analysis of viral DE miRNA targets. The horizontal axis represents fold enrichment; the vertical axis represents significant pathways (*Q* < 0.05); the size of solid circles represents gene number in each pathway; and ColorRamp represents enrichment significance.

Animal-type target gene analysis with stringent criteria against the de novo transcriptome yielded 8237 genes targeted by host DE miRNAs and 3886 genes as the targets of viral miRNAs. KEGG enrichment analysis showed that these target genes were significantly enriched in a wide variety of metabolic pathways (Fig. 3). For host miRNAs, lipid-related metabolism, such as glycerolipid metabolism, sphingolipid metabolism, and FA elongation, were significantly enriched (Fig. 3A). We also found that steroid biosynthesis, terpenoid backbone biosynthesis, sphingolipid metabolism, autophagy, etc. were targeted by viral miRNAs (Fig. 3B). The high expression of viral miRNAs at the late infection stage might regulate host metabolism to meet the special metabolic requirements of viruses or prevent the premature of host autophagy [30]. Target genes of host miRNAs were also enriched in the PI3K-Akt signaling pathway. In multicellular organisms, the PI3K-Akt signaling pathway combined with its downstream pathway (e.g., mTOR signaling pathway, which was also significantly influenced according to our transcriptomic data) played an important role in regulating FA and glycerolipid metabolism [51, 57, 58]. We speculated that lipid metabolism in the *E. huxleyi*–EhV system might be regulated via the miRNA-targeted PI3K-Akt signaling pathway. Therefore, further proofs of this speculation are explored in the following sections.

### *PI3K* is a potential target of ehx-miR5 in the CDS region

**Fig. 4:**
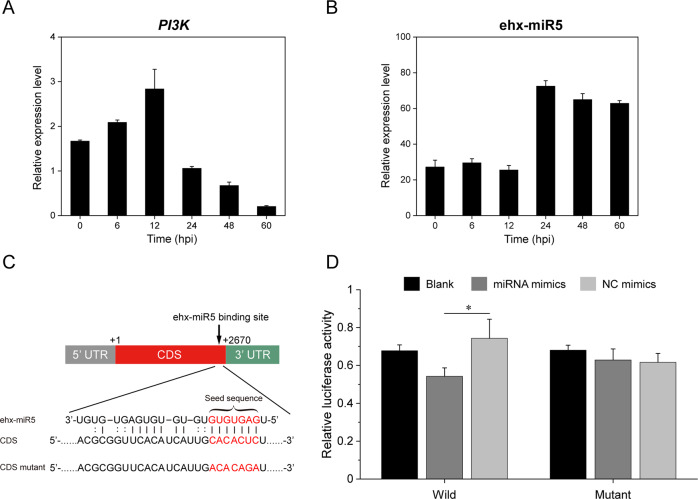
Targeting relationship analysis between *PI3K* and ehx-miR5. **A**, **B** qRT-PCR results of *PI3K* (**A**) and ehx-miR5 (**B**). **C** Putative binding sites of ehx-miR5 on the CDS region of *PI3K* predicted by miRanda; mutations are written in red. **D** The relative luminescence ratio in the *PI3K* CDS luciferase reporter assay. Blank groups were used as controls. The results were analyzed by Student’s *t* test. *p < 0.05.

Based on the bioinformatic prediction of miRNA targets in virus-infected *E. huxleyi*, we found *PI3K* gene was targeted by ehx-miR5 with perfect pairing on the CDS region by screening our de novo transcriptome database. BLASTn analysis of the *PI3K* fragment based on the genome of *E. huxleyi* CCMP1516 revealed that no 3′-poly A tail was detected in the *PI3K* fragment. Therefore, the complete CDS and 3′-UTR regions of *PI3K* were acquired by the 3′-RACE approach, but there was no target site on the 3′-UTR (data not shown). A previous study demonstrated that endogenous miRNAs in the green alga *C*. *reinhardtii* predominantly regulated gene expression through CDS-targeting [59]. Moreover, the expression levels of ehx-miR5 and *PI3K* were both verified by qRT-PCR and they displayed a negative correlation (the Pearson correlation coefficient was −0.837) (Fig. 4A, B). Thus, a dual-luciferase reporter assay was employed to further validate the prediction. The coding sequences of *PI3K* were cloned into the psi-CHECK2 vector to construct a recombinant vector (wild-type vector, WT vector) and mutations were made in the CDS seed sequences to construct another vector (mutant type vector, MT vector) (Fig. 4C). The constructed vectors were identified by restriction enzyme digestion (Supplementary Fig. S19) and nucleotide sequencing. The dual-luciferase reporter assay showed that relative luciferase activity significantly decreased by 27.1% in *PI3K* group compared with the control mimics (*p* < 0.05), and the results were not significantly different for transfection with the mutant vector (Fig. 4D). These results indicated that *PI3K* gene might be a target of ehx-miR5.

### miRNA-targeted PI3K-Akt-TOR signaling pathway is inhibited during late infection

**Fig. 5:**
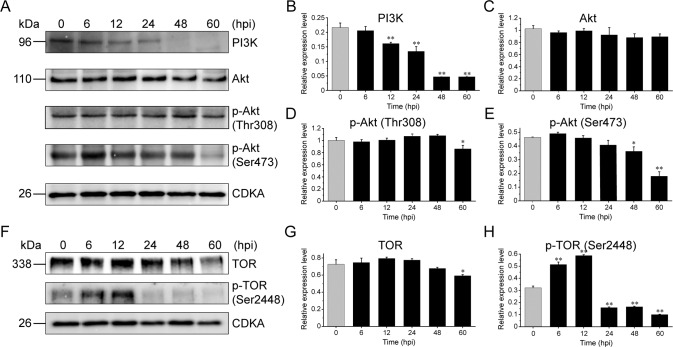
Protein expression levels of components in the PI3K-Akt-TOR signaling pathway during EhV infection. **A**–**E** Representative bands by western blot for PI3K, Akt, p-Akt (Thr308), and p-Akt (Ser473) (**A**) and their quantitative analysis by ImageJ (**B**–**E**). **F**–**H** Representative western blots of TOR and p-TOR (Ser2448) (**F**), and their quantitative analysis (**G**, **H**). *p < 0.05, **p < 0.01.

In animal cells, the PI3K-Akt-mTOR signaling pathway has been proven to regulate lipid biosynthesis by promoting the nuclear accumulation of the mature form of SREBP [51, 57], a nuclear transcription factor that can promote the transcription of acetyl-CoA carboxylase (*ACC*), fatty acid synthase (*FAS*) and glycerol-3-phosphate O-acyltransferase (*GPAT*) [57, 60, 61]. Despite the important role of the PI3K-Akt-TOR signaling pathway in lipogenesis, its regulatory mechanism in phytoplankton (especially in virus-infected *E. huxleyi*) remains largely unexplored. Based on the de novo transcriptomic data, a complete gene annotation of the PI3K-Akt-TOR signaling pathway was obtained, facilitating the subsequent verification by western blotting and quantitative analysis (Fig. 5). The protein expression levels of PI3K gradually decreased starting from 12 until 60 hpi compared with the uninfected group (0 hpi) (Fig. 5A–E). The levels of Akt phosphorylation at Ser473 decreased significantly at 48 and 60 hpi, while the decreased phosphorylation of Thr308 was only observed at 60 hpi, concurrent with no significant effect on the total Akt levels (Fig. 5A–E). The phosphorylation levels of the downstream TOR at the Ser2448 site showed a significant increase at 6 and 12 hpi and then drastically decreased at 24–60 hpi, while only at 60 hpi did the total TOR levels moderately decrease (Fig. 5F–H), indicating that TOR signaling was impeded during late viral infection. These observations suggest that the PI3K-Akt-TOR signaling pathway is activated during early infection and inhibited during late infection.

### Viral infection regulates the nucleoprotein levels of lipin 1 and SREBP

**Fig. 6:**
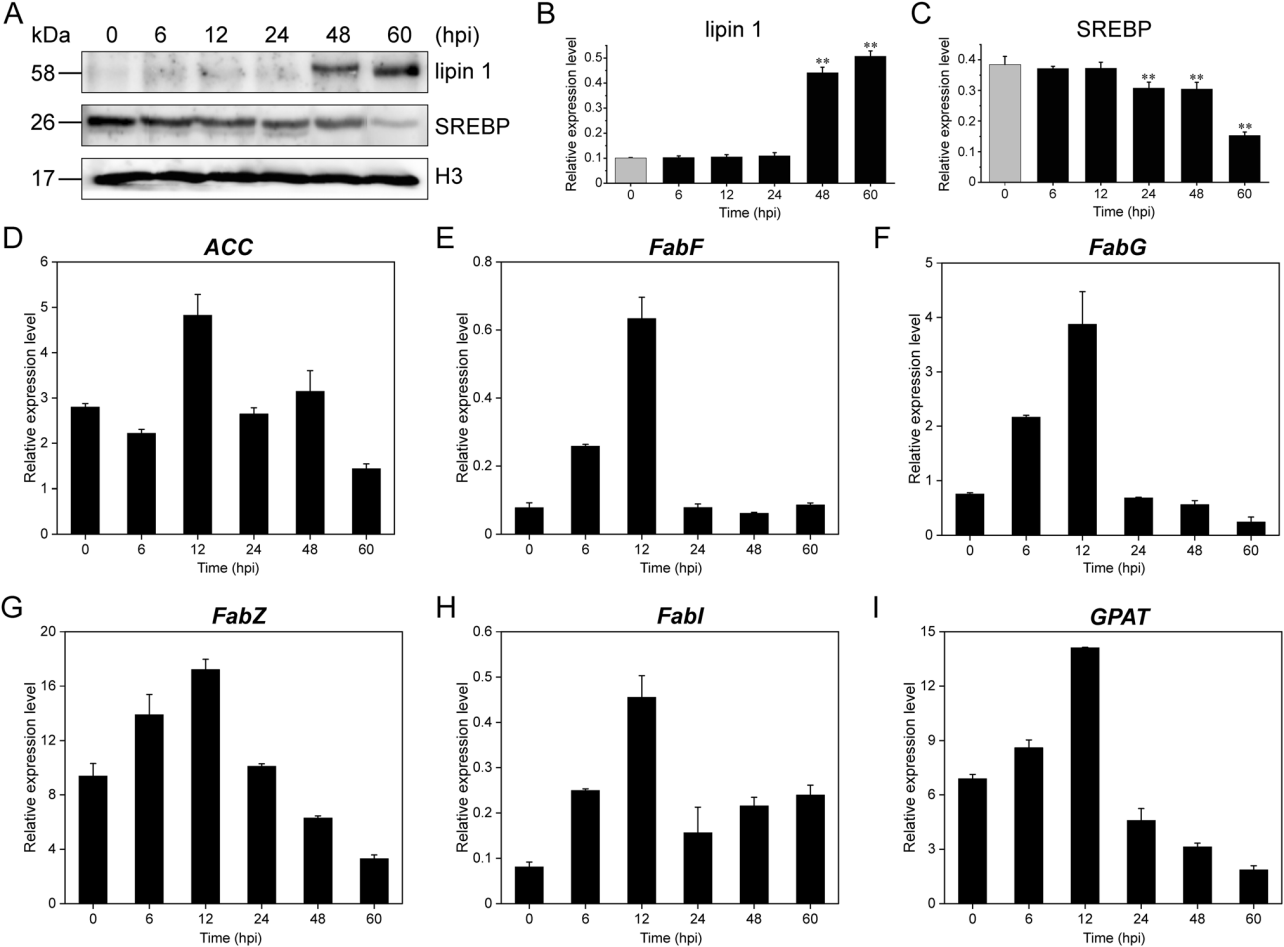
Expression levels of nucleoprotein lipin 1, SREBP, and the downstream genes mediated by SREBP. **A** Representative bands for lipin 1 and SREBP and **B**, **C** indicate their quantification. **p < 0.01. **D**–**I** qRT-PCR results of SREBP-mediated genes.

It has been demonstrated that activated TOR can phosphorylate lipin 1, a phosphatidic acid phosphatase, and phosphorylated lipin 1 cannot enter the nucleus to reduce SREBP promoter activity and nuclear SREBP protein abundance [58]. Based on the experimental results above, we speculated that the expression of the lipogenesis-related transcription factor SREBP and genes related to lipid synthesis was inhibited during EhV infection. We therefore examined the levels of intracellular nucleoprotein lipin 1 and SREBP by western blotting. The expression levels of lipin 1 increased markedly at 48 and 60 hpi, exhibiting a completely opposite trend with SREBP levels (Fig. 6A–C). The high expression levels of SREBP in early infection (0–12 hpi) favored the lipid-related gene transcription, including *ACC*, *FAS*, and *GPAT*, as verified by qRT-PCR (Fig. 6D–I), which further promoted TG accumulation during early EhV infection (Supplementary Fig. S13). Therefore, we believe that the expression of lipogenesis-related enzymes is regulated by TOR via lipin 1 and SREBP.

### The potential miRNA regulatory mechanism for lipid metabolism in the *E. huxleyi*–EhV system

**Fig. 7:**
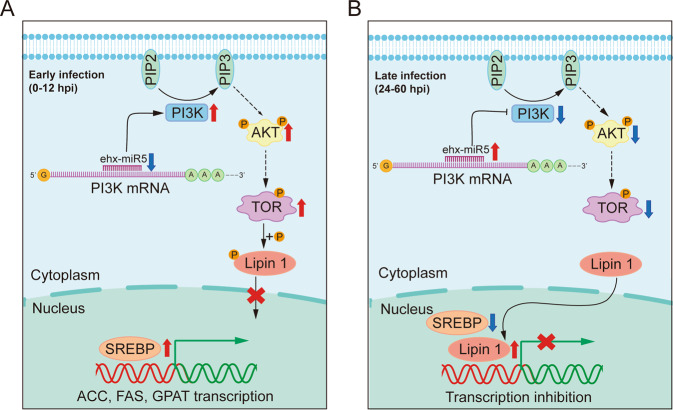
Schematic of miRNA-targeted lipid metabolism in the *E. huxleyi*–EhV system. During early infection, low expression levels of ehx-miR5 lead to high expression of PI3K. The activated PI3K-Akt-TOR signaling prevents the entry of lipin 1 into the cell nucleus by phosphorylation, and hence SREBP can promote gene transcription (**A**). In contrast, during late infection, PI3K-Akt-TOR signaling is inhibited under the effect of ehx-miR5. Dephosphorylated lipin 1 enters the cell nucleus and decreases the abundance and activity of SREBP (**B**).

Based on the results above, we concluded a potential miRNA-based regulatory mechanism for lipid metabolism through the PI3K-Akt-TOR signaling pathway in virus-infected *E. huxleyi* cells (Fig. 7). During early infection (0–12 hpi), the relatively low expression of ehx-miR5 resulted in high expression of PI3K, which facilitated the activation of Akt by phosphorylation. Relying on the activated Akt, TOR was phosphorylated and then phosphorylated lipin 1, preventing the entry of lipin 1 into the cell nucleus. As a result, SREBP promoted genes transcription (Fig. 7A). During late infection (24–60 hpi), the high expression of ehx-miR5 led to low levels of PI3K, suppressing the phosphorylation of Akt, TOR, and lipin 1, and finally lipin 1 entered the cell nucleus, causing decreased levels of SREBP abundance and activity (Fig. 7B). Overall, these observations are basically in line with the expectation that the animal-like regulatory mechanism of lipid metabolism possibly exists in virus-infected *E. huxleyi* through PI3K-Akt-TOR-lipin 1-SREBP in an orchestrated manner.

The regulatory functions of miRNAs in lipid metabolism in animals are widespread and conserved processes. They cannot only regulate hub genes that directly participate in lipid metabolism, such as FA biosynthesis and degradation, and glycerolipid biosynthesis, but also indirectly mediate lipid metabolism through activating signal pathways (such as AMPK and PPARα signaling pathways) [21, 25, 27, 62]. miRNA (miR-378) can target p110α, a catalytic subunit of PI3K and hence a key transducer of the insulin signaling pathway, and thus controls glucose and lipid homeostasis through enhancing the liver’s response to feeding/fasting cues mediated by insulin [63]. Besides, some ATP-binding cassette (ABC) transporters are repressed by miRNAs, reducing the efflux of lipids from cells. Virus-derived miRNAs identified in our study were also predicted to target ABC transporters (Fig. 3B), indicating a potential strategy for the virus to accumulate lipids in *E. huxleyi* cells [64–66]. In this study, due to the limitation of experimental conditions, we were not able to directly validate the miRNA’s regulatory effect on PI3K by stable transfection in *E. huxleyi* cells, but we are confident that the results of our work are meaningful for exploring the miRNA-targeted lipid metabolism in marine phytoplankton infected by viruses.

## Conclusions

We applied lipidomic, transcriptomic approaches, and experimental verification to reveal the reprogrammed lipid metabolic responses to viral infection in the coccolithophores *E. huxleyi* that are regulated at transcriptional, miRNA-mediated posttranscriptional, and translational levels. Moreover, our results demonstrate the possible regulatory role of miRNA in lipid metabolism through the PI3K-Akt-TOR signaling pathway. In addition, our findings also further indicated that cellular calcium ions might be important for maintaining lipid homeostasis in virus-infected *E. huxleyi* cells. These findings shed new light on the epigenetic regulation between marine algae-virus interactions and enable us to deepen the understanding of the chemical “arms race” among marine microbes.

## Supplementary information

Supplementary information

Supplementary Dataset 1

Supplementary Dataset 2

Supplementary Dataset 3

Supplementary Dataset 4

Supplementary Dataset 5

## Data Availability

The generated raw data of the de novo transcriptome were deposited to NCBI in SRA (BioProject accession number: PRJNA705400). The Transcriptome Shotgun Assembly (TSA) project has been deposited at DDBJ/EMBL/GenBank under the accession GJZP00000000. The version described in this paper is the first version, GJZP01000000. The lipidomics data have been deposited to the EMBL-EBI MetaboLights database with the identifier MTBLS5178.
