# Supplementary material for: MicroRNA-mediated regulation of lipid metabolism in virus-infected Emiliania huxleyi
Source: ISME J. 2022 Jul 22;16(11):2457–66. doi: 10.1038/s41396-022-01291-y (PMC9561107; doi:10.1038/s41396-022-01291-y)
Supplement: Supplementary file 1 — Supplementary information [file 41396_2022_1291_MOESM1_ESM.pdf]

Supplementary Information

MicroRNA-mediated regulation of lipid metabolism in virus-infected *Emiliania huxleyi*

Enquan Zhang, Jingjing Gao, Zehua Wei, Jun Zeng, Jian Li, Guiling Li*, Jingwen Liu*

College of Ocean Food and Biological Engineering, Jimei University, Xiamen, 361021, China

* Corresponding author:

Prof. Jingwen Liu, Tel.: 86-592-6181487. E-mail: ljwsbch@163.com

Dr. Guiling Li: glli2016@jmu.edu.cn

Pages: 44

Figures: 19

Tables: 10

Datasets: 5

Supplementary information Appendix

Supplementary Materials and Methods

Enumeration of cell and virus abundance

Transmission electron microscopy (TEM) analysis

Lipid internal standards

Intracellular reactive oxygen species (ROS) detection

Mitochondrial membrane potential (MMP) detection

Intracellular ATP detection

Ca^{2+} flux measurement

Nucleoprotein extraction

Supplementary Results

Quality evaluation and global changes of lipid profiling

Reprogrammed lipid metabolism during viral infection

Supplementary Tables:

Table S1. Oligonucleotide primers used for mRNA qPCR.

Table S2. Oligonucleotide primers used for miRNA qPCR.

Table S3. List of antibodies used in this study.

Table S4. Illumina sequencing statistics.

Table S5. Assembly quality statistics.

Table S6. Overview of all unigene annotation.

Table S7. Assignment of the identified metabolites to lipid classes and amount of lipid species identified in this study.

Table S8. Annotation and accession numbers of unigenes verified by qPCR.

Table S9. Differentially expressed miRNAs in terms of log2 (fold change) from the transcriptome and -ΔΔCt from qPCR.

Table S10. Plant-type targets of host miRNAs which were related to lipid metabolism.

Supplementary Figures:

Figure S1. Infection dynamics, ultrastructure, and neutral lipid accumulation during EhV infection.

Figure S2. Gene annotation of the *de novo E. huxleyi* transcripts

Figure S3. Relationship analysis of samples.

Figure S4. Weighted gene co-expression network analysis (WGCNA).

Figure S5. Base peak chromatography of total lipid profile.

Figure S6. Quality evaluation of total lipid profile.

Figure S7. PLS-DA model of all samples.

Figure S8. Global lipidomic profiles and pathway enrichment analysis during the large-scale infection process.

Figure S9. Heatmap visualization of total lipid profile.

Figure S10. The hierarchical heatmap of all 443 viral genes.

Figure S11. The qRT-PCR results of selected genes relevant to lipid metabolism.

Figure S12. Integrated metabolic map of fatty acid biosynthesis and β-oxidation.

Figure S13. Integrated metabolic map of glycerolipid metabolism and glycerophospholipid biosynthesis.

Figure S14. Integrated metabolic map of host and viral sphingolipid metabolism.

Figure S15. Detection of mitochondrial functions and Ca^{2+} flux during EhV infection.

Figure S16. The hairpin structures of host and viral miRNAs.

Figure S17. The regression analysis of gene expression between the results of qRT-PCR and miRNA-seq.

Figure S18. Gene annotation of the plant-type targets of host miRNAs.

Figure S19. The agarose gel electrophoresis results of restriction enzyme digestion of each recombinant psi-CHECK2 vectors.

Supplementary Datasets:

Supplemental Dataset 1. List of putative lipids identified in *Emiliania huxleyi.*

Supplemental Dataset 2. Abbreviations of gene names used in Figures S12-14.

Supplemental Dataset 3. The filtration of small RNA reads.

Supplemental Dataset 4. Details of miRNAs identified in this work.

Supplemental Dataset 5. Differential expression of miRNAs in different comparative groups.

Supplementary Materials and Methods

Enumeration of cell and virus abundance

The sample analysis was performed with an Epics Attra II flow cytometer (Beckman–Dickinson) equipped with an external quantitative sample injector (Harvard Apparatus PHD 2000) and a water-cooled laser providing 0.100 ± 0.01W at 488 nm. For enumeration of algal cells, frozen samples were quickly thawed and run with an injection flow rate of 50~100 events s^{-1} and with the discriminator set on red fluorescence. Virus enumerations were performed on frozen samples that were quickly thawed, diluted from 1:10 to 1:1000 in TE buffer (10mM Tris, 1mM EDTA, pH 8.0), and stained for 10 min at 80°C with SYBR Green-1 (Molecular probes, Eugene, OR, USA) at a final concentration of 10^{-4} of the commercial solution. The samples were analyzed at a flow rate of 50-100 events s^{-1} with the discriminator set on green fluorescence.

Transmission electron microscopy (TEM) analysis

Fixed cells were washed three times with 0.1 M PBS (pH 7.4) and post-fixed for 1 h in 1.0 % osmium tetraoxide at 4°C. Samples were washed in buffer, dehydrated in graded series of ethanol and embedded in Epon. Sections were cut using an LKB 2088 ultramicrotone, collected on 200-mesh copper grids, and stained with uranyl acetate and lead citrate. The stained sections were photographed with a JEM-100CXII electron microscope.

Lipid internal standards

The lipid internal standards used for lipidomic analysis were as follows: 1.5 mg/mL of FA (16:0). 1.5 mg/mL of FA (18:0), 1.3 mg/mL of LysoPC (19:0), 2.8 mg/mL of PC (38:0). 1.1 mg/mL of PE (30:0), 1.3 mg/mL of SM (12:0), 1 mg/mL of TG (45:0), 0.7 mg/mL of Cer (17:0). Methanol (HPLC grade) was

used as solvent and the internal standard solutions were stored at 20℃.

Intracellular reactive oxygen species (ROS) detection

10 ml algal cultures were centrifugally collected (4℃, 3000 rpm, 5 min) and resuspended using f/2-si medium. Experiment groups were set up as follows: control groups (*E. huxleyi*); infection groups (*E. huxleyi* + EhV); positive groups (*E. huxleyi* + Rosup). ROS was measured with the reactive oxygen species assay kit (Beyotime, Shanghai, China) according to the manufacturer's instructions. Briefly, resuspended algal cells were incubated in f/2-si medium containing 10 mM 2'-7'-dichlorofluorescein diacetate (DCFH-DA) fluorescent probes at 16°C for 30 min in the dark and then washed with 1 mL PBS three times to remove residual probes (centrifugally collected at 4°C, 3000 rpm, 5 min). Then cells were resuspended with 500 μL PBS and DCF fluorescence was detected by FACScan flow cytometer (Becton Dickinson) at 488 nm excitation wavelength and 525 nm emission wavelength.

Mitochondrial membrane potential (MMP) detection

Sample collection procedures and experiment groups were basically consistent with the ROS detection except that the positive control was carbonyl cyanide 3-chlorophenylhydrazone (CCCP). The change of MMP in *E. huxleyi* cells was monitored with the MMP Detection Kit with JC-1 (Beyotime, Shanghai, China). The algal cells were harvested and treated with a JC-1 working solution (5 μg mL^{-1}) for 20 min at 16°C in the dark. After washing in cold JC-1 staining buffer, the fluorescence intensity of the cells was monitored at excitation/emission wavelengths of 514/529 nm (JC-1 monomers) and 585/590 nm (JC-1 aggregates) using FACScan flow cytometer (Becton Dickinson). Aggregate/monomer (red/green) fluorescence intensity ratio was used to quantify MMP.

Intracellular ATP detection

The ATP levels in cells were detected using an ATP assay kit (Beyotime, Shanghai, China) according to

the protocol provided by the manufacturer. In brief, cells were gathered with centrifuging for 5 min at 4°C, 3000 rpm and lysed in the lysis solution, collecting supernatant after centrifuging for 5 min at 4°C, 12,000 rpm. ATP detection reagent was diluted to one tenth with ATP detection reagent dilution, incubated for 35 min at room temperature. Then 100 μL ATP detection working fluid were mixed with 20 μL prepared standards and samples separately in 96-well plate. The results were detected by a microplate reader (Beckman Coulter, USA).

Ca^{2+} flux measurement

A platinum microelectrode and Ca^{2+}-selective microelectrode in the NMT (Non-invasive Micro-test Technology) System for Live Samples (Younger USA LLC., Amherst, MA, USA) were used to measure the real-time Ca^{2+} flux of *E. huxleyi* cells by Xuyue Science and Technology Co. (Beijing, China). Sample pretreatment: 35 mL of algal cultures were centrifugated at 3000 rpm for 3 min and then resuspended by 700 μL of measuring solutions (2.0 mM CaCl2, 400 mM NaCl, 50 mM MgCl2, pH 9.0). Ca^{2+} flux measurement: 350 μL of resuspended samples were added into a conical container (diameter 10 cm, made by a 0.45 μm filter membrane), the cone tip of which was fixed into a 6 cm-diameter dish. Then 10 mL measuring solutions were added into the dish to ensure the cone tip was absolutely immersed. The Ca^{2+} flux microsensor was placed at 10 μm from the cone tip and the fluxes were recorded every 6 secs by software imFluxes V2.0 (Xuyue Company, Beijing, China). Non-infected cells were firstly measured. The measurement time points were set up at 0 h (non-infected), transient infection (0–20 mins post infection), 2 hpi, 12 hpi and 24 hpi, respectively. Continuous testing at each time point were lasted for 5 mins except the transient infection (lasting for 20 mins). Each measurement was set up as 12 replicates.

Nucleoprotein extraction

The extraction and isolation of nuclear and cytoplasmic protein were performed according to the Nuclear

and Cytoplasmic Protein Extraction Kit (Beyotime, Shanghai, China). First, 200 mL of algal cultures were centrifuged for 5 min at 8000 rpm at 4°C and the pellet was dissolved with 400 μL of cytoplasmic protein extraction agent A supplemented with PMSF. After vortex for 30 s, the tubes were incubated for 15 min on ice to promote lysis. Next, add 20 μL of the cytoplasmic protein extraction agent B, vortex for 5 s and incubated on ice for 1 min. Then the samples were centrifuged for 5 min at 13,500 rpm at 4°C and the supernatant, consisting of the cytosolic fraction, was immediately frozen for further analysis. The pellet was resuspended in 50 μL of nuclear protein extraction agent supplemented with PMSF. After vortexing the tubes 15-20 times for 30 min and centrifuging for 10 min at 13,500 rpm at 4°C, the supernatants containing the nuclear extracts were obtained.

Supplementary Results

Quality evaluation and global changes of lipid profiling

Nontargeted lipidomic analysis was performed to explore host-virus interactions. The large-scale lipid profiling of *E. huxleyi* revealed approximately 500 lipid features (Fig. S5). A total of 246 lipid species were identified (Supplemental Dataset 1), including 53 phospholipids (PLs), 26 sphingolipids (SLs), 144 glycerolipids (GLs), 20 FAs, 2 wax esters (WEs) and 1 of CmE (Table S7). The quality of the resulting lipid profiling was examined by evaluating quality control (QC) samples and was confirmed to be satisfactory for complex biological samples according to our published methods [1] (Fig. S6). Prior to subsequent analysis, lipids with a percentage relative standard deviation (%RSD) higher than 30% in all QCs were removed from the dataset. An overview of *E. huxleyi* lipidome deregulation upon viral infection was visualized using a multivariate partial least squares discriminant analysis (PLS-DA) model, and six types of metabolic disturbance induced by viral infection were clearly visible on the score plot along the first principal components (Fig. S7A). The PLS-DA model was validated without overfitting based on response permutation test with 200 iterations (Fig. S7B).

The relative levels of all lipid metabolites were scaled by z score and subjected to hierarchical clustering heatmap to visualize the variation tendencies during viral infection. These lipid classes were clustered into four major change models (clusters 1-4) (Fig. S8A), and the lipids from different clusters were subsequently subject to KEGG enrichment analysis (Fig. S8B-E). Moveover, the sum of lipid levels was also visualized by a plotted heatmap (Fig. S9). The level trends of lipids from cluster 1 gradually decreased with the duration of infection, while the lipid levels in cluster 2 were upregulated during 6-24 hpi. The lipids in cluster 1 and cluster 2 were both significantly enriched in glycerophospholipid, sphingolipid and glycerolipid metabolism (Fig. S8B, C), specifically including most PLs (PSs, PEs, PIs,

PAs, PGs, and CLs), host SLs (hCers, hGSLs, and SMs) and GLs (MGDGs, DGs, and TGs) (Fig. S9). Lipids related to the biosynthesis of unsaturated FA, the most significant pathway (Fig. S8D), were mainly from cluster 3, which exhibited a downregulation at 12 and 24 hpi (Fig. S8A, Fig. S9). Cluster 4 predominantly contained most TGs, viral Cers (vCers) and vGSLs which were upregulated at 48 and 60 hpi (Fig. S8A, Fig. S9), corresponding with the significant pathways enriched in cluster 4 (Fig. S8E). The diversity of metabolic disruptions during virus infection implied complicated dynamics of the host-virus interaction.

Reprogrammed lipid metabolism during viral infection

FA metabolism-The expression patterns of genes and metabolites related to FA biosynthesis were strongly affected by EhV infection (Fig. S12). Although the expression trends of acetyl-CoA carboxylase (ACC), the rate-limiting enzyme of FA biosynthesis, was fluctuant, it reached the highest expression level at 12 hpi (1.72-fold compared with that at 0 h). Simultaneously, most of the FA synthase except FabD also showed a trend towards higher expression in early infection (0-12 hpi). However, two types of free saturated FAs (FA 16:0 and 18:0) detected in our lipidome data both gradually decreased during infection (Fig. S12). This might be explained by the following reasons. First, we found that nearly all genes participating in host β-oxidation (except ACADL) were highly expressed in early stage (0-12 hpi), which might result in high decomposition efficiency to FAs (Fig. S12). Second, long chain acyl-CoA synthase (ACSL), which catalyzes the acylation of long chain FAs, was gradually upregulated and reached its maximum at 24 hpi (3.02-fold), further consuming FAs to synthesize other types of lipids (Fig. S12). In addition, we speculated that some continuously increased unsaturated FAs (FA 20:4, 16:1 and 20:5) were transformed from saturated FAs (Fig. S12). These observations indicated that there was a high demand for FAs during the early phase of viral infection.

Glycerolipid metabolism-The metabolic map of glycerolipid metabolism (including glycerophospholipid metabolism) was shown in Fig. S13. Almost all genes involved in glycerolipid biosynthesis were induced and reached their maximal levels during early infection, such as glpK (3.05-fold at 12 hpi), GPAT (2.01-fold at 12 hpi), hLPP (2.10-fold at 12 hpi) and DGAT (2.99-fold at 6 hpi) (Fig. S13). Simultaneously, the high expression of vLPP (851.76-fold higher at 48 hpi than at 24 hpi) made it possible to promote glycerolipid biosynthesis at late infection stage (Fig. S13). In agreement, the metabolic levels of sum of triacylglycerols (TGs) were elevated during the whole infection process, reaching the top at 60 hpi (Fig. S13). This increased abundance of TGs coincided with a decrease in the relative abundance of monogalactosyldiacylglycerols (MGDGs) and diacylglycerols (DGs) during infection (Fig. S13). Despite the increased profiling of sum of TGs, the abundance of partial TGs showed a gradual decrease during the infection process (Fig. S8A) concomitantly with a significant upregulation of host TGL (hTGL, 2.70- and 2.24-fold at 12 and 24 hpi, respectively) and viral TGL (vTGL, 701.91- and 1887.55-fold higher at 48 hpi and 60 hpi than at 24 hpi, respectively) (Fig. S13). We speculated that this part of TGs was hydrolyzed by TGL to release FAs that could be oxidated to generate energy for viral production, which might account for the increase of some unsaturated FAs (Fig. S13). The abundance of PIs was decreased compared with the control group (1.24-1.69-fold decrease) (Fig. S13). It was assumed that most PIs were phosphorylated to generate PIP, PIP2 or PIP3, which served as signal molecules playing a prominent role in virus entry [2]. Other PLs were gradually increased during the infection, reaching the maximum levels at 12 hpi (PG and PE), 24 hpi (CL) or 48 hpi (PC).

Sphingolipid Metabolism-The expression pattern of genes (including viral genes) related to sphingolipid metabolism was strongly consistent with the abundance of sphingolipid metabolites (Fig. S14). During early infection stage (0-12 hpi), host genes participating in de nove sphingolipid

biosynthesis except 3-ketodihydrosphingosine reductase (KSR) showed a high expression, corresponding to the abundance of the detected SLs, including dihydroceramide and ceramide, which were significantly accumulated in early infection stage (Fig. S14). Reversely, during the late infection stage (24-60 hpi), the host expression in both transcriptional level and metabolite level were sharply decreased (Fig. S14). Other deviants of sphingolipids like glucosylceramide and sphingomyelin exhibited a similar expression trend (Fig. S14). In contrast to host sphingolipid expression patten, all viral genes participating in de novo sphingolipid biosynthesis, such as serine palmitoyltransferase (SPT), dihydroceramide desaturase (DCD), and ceramide synthase (CerS) showed a profound overexpression (210-1668-fold increase compared with 24 hpi) at the late stages (48 hpi and 60 hpi) of infection (Fig. S14). In addition, virus-encoded FA hydroxylase (vFAH) was highly expressed at the late stage of infection (Fig. S14), which hydroxylases the FA chain bases of dihydrosphingosine or ceramide to generate phytosphingosine or phytoceramide, respectively. Conformably, virus-specific ceramides and glucosylceramides also showed extremely high abundance at 48 and 60 hpi and contained hydroxylated FA chain bases (Fig. S13). Besides, according to the previous study [3], vSPT could make use of C14-CoA or C15-CoA as substrates to produce ceramides, which is consistent with the C16 or C17 FA chains contained in viral phytoceramide from our data (Fig. S14). Taken together, these observations reveal the inhibited host sphingolipid metabolism and a metabolic shift towards viral sphingolipid metabolism during EhV infection in *E huxleyi*-EhV system.

Reference

1. Zeng J, Liu S, Cai W, Jiang H, Lu X, Li G, et al. Emerging lipidome patterns associated with marine *Emiliania huxleyi*-virus model system. Sci Total Environ. 2019; 688: 521–528.
2. Mazzon M, Mercer J. Lipid interactions during virus entry and infection. Cell Microbiol. 2014; 16:

1493–1502.

3. Ziv C, Malitsky S, Othman A, Ben-Dor S, Wei Y, Zheng S, et al. Viral serine palmitoyltransferase induces metabolic switch in sphingolipid biosynthesis and is required for infection of a marine alga. Proc Natl Acad Sci USA. 2016; 113: E1907–E1916.

Supplementary Tables

Table S1. Oligonucleotide primers used for mRNA qPCR.

Gene	Forward primer sequence (5'→3')	Reserve primer sequence (5'→3')
ACC	TTCAAGGTGGCCTGGAACG	GGTGATGTACTCGCCGTGGAA
FabF	CGAACTACGGCATCGTGTCTG	TCGCCCATCACGAAACCC
FabG	GAGGAGTGGGACGCTGTGAT	CGACCGCATGAAACCGA
FabZ	AAGGCGATTTCTTCTTTGCG	CGACACCCTTCATTTTGACCAG
FabI	GACATCCTGGTCCACTCCCT	CCTTCTCCGAAGCGATGTAC
CAT2	CAGGACTCGCTGCCCAAGAT	TGCGACACCTTTGGCTTTGT
ACOX	GTCCGCTTCACCGTCCAGTT	CGACGATCAGTCCAGACAGCA
ACADS	GTCGGTCAACAACTCGCTCTA	CGCCTTTGTGCCCTCTAAC
ACAT1	CGTCCAACCAGGCGATTT	GAGGTCCTTCATCTTCAGTCCC
glpK	CGTCGCTCATCGCATCCAT	CCTTTTGGCGTCCATCTGC
GPD	CACGTCCGTGACTGACATCTG	ACGCCACATTGCCACCATT
GPAT	TCTCAAGAGCCTGGTCTGCT	GCCGTCAGGAAGTGGATGT
LPP	CTTTGTCTCTGTGGGCGTGTG	CGTCCAAGTCGTCGTGTTCG
TGL	TCGTGCTATCCGTTCTGCG	TTGGATACCCAGGCAAAGGA
MGLL	TGCGACTGCTGGTGCTATG	GGAGGTCGTCGATGTTGGA
SPT	TCTCGGACACGCTCAACCA	CCCTCGACGATGATGATGATC
KSR	ATCTGGCCGGAGATGTTCA	CATCCAGACCATCACGCAGT
CerS	GAGGAGCGGCACAAGGACTAT	CCGATGCGGACAAAGTTGAA
DCD	CGGAGTGGCGGTCAAAGTA	CGGCGACTTGAAGAAGAGGT
UGCG	CCCGTCTCCTTCCTCTTTGT	TGAGGTTGTGCAGCTTCTGG
GBA	GGCTCAACACGGAGAACCTG	GGTTCTTGAGGTGGTTCGGTC
SPK	TCTGTGAGCATTAAGACGGAGA	ACGAAGCAGGAGAAGAGGAAG
PI3K	CCATTTCCTGGGCAACTACA	CAGCAGAGGGCTACGAAGTG
AKT	CCACTTCCTCACCGACATCAA	TGATGGCGGTCCCGAAGA
TOR	CAGCACAATCTCCTTCTGCAC	TGCCGATTTGCGGGTAC
vSPT	ACGAAAAGTGTCGGGCTACG	TGAATGTCAAGCGTCCCGT
vCerS	AATCCAGGAAGCGGTATCAA	CATCAAGTCGTTCGTCTCCAA
vDCD	AAAGAACAACCGATAGACACCG	GGGATTGAATGACGATTAGGAGT
vFAH	ATACAACAATGGGGAAGTCGC	AAGTAATCCACTGTTGCTCCGT
vLPP	TCCATCGGGTCATTCTTCG	CAGGTGGATGATGCCAGAAA
vTGL	ACATTTGGTTCATCACGCACAG	TCTAACGCCACGCCAATTTC

Table S2. Oligonucleotide primers used for miRNA qPCR.

Small RNA ID	Primer sequence
U6-F	CTCGCTTCGGCAGCACA
U6-R	AACGCTTCACGAATTTGCGT
ehx-miR1-5p	GGTCACACCTGCAGCTGT
ehx-miR2-3p	CGGGCGTGTGAGAGAGAGAGATTTA
ehx-miR3-3p	CTGAGGATGACGGCGTTGAGC
ehx-miR4-5p	AGGTCGATCGTTGGCACCAAA
ehx-miR5	TGAGTGTGTGTGTGTGAGTGTGT
ehx-miR6-3p	AACTTGGAGCACTTGGAGTGCAG
ehx-miR7-5p	CTACGGCACCGCGTGGGA
ehx-miR8-3p	CGTGAATCGTGTCGTGCGACT
ehx-miR9-5p	GAGGCATTCGCATCGTGATATG
ehx-miR10-3p	AGATGACTGTGCGTGACGCG
ehx-miR11-3p	GCGGAATTCCAATGGGGAGTAA
ehx-miR12-5p	GTTACAAGGGACGCACTGCAAA
ehx-miR13-3p	GGAGGCTGGCTCGCTCGC
ehx-miR14-3p	CGAGATCGTCGAGATGCGCC
ehx-miR15-3p	CGCTCGTCGGCGGCAAAA
ehx-miR16-3p	GCCACCTCCGCCGCCTAA
ehx-miR17-5p	GGCTGCAAGGTGGACGCC
ehx-miR18-5p	CTCTCGGGCGGCCAGAAGC
ehx-miR19-5p	TCGACAAGGACGGCGACGG
ehx-miR20-5p	CGGCGGTAGTCGGCGGTAAA
ehv-miR1-3p	GGAATTGGTCGTCACGTTGTTGT
ehv-miR2-3p	GGTGAGAGTGCATCGGATTGTGAA
ehv-miR3-3p	GGATTTGGGCTGGCCCAAAA
ehv-miR4-3p	GGATCTAGGAAAGATTGAGGCCAAA
ehv-miR5-3p	GGCGAAGACACTGTGAATCAAGT
ehv-miR6-5p	CGGGCGGAAAATATGATTCGTTA
ehv-miR7-5p	GCCTGACGCGGATACTGTCTC

Table S3. List of antibodies used in this study.

Name	Antibodies information	Manufacturer	Product number	Dilution ratio
PI3K	PI3 Kinase p110α/PIK3CA Rabbit Polyclonal Antibody	Beyotime, China	AF7749	1：1000
Akt	AKT1/2/3 Rabbit Monoclonal Antibody	Beyotime, China	AF1789	1：1000
p-Akt (Thr308)	Phospho-AKT1 (Thr308) Rabbit Polyclonal Antibody	Beyotime, China	AF5734	1：1000
p-Akt (Ser473)	Phospho-AKT1 (Ser473) Rabbit Polyclonal Antibody	Beyotime, China	AF5740	1：1000
TOR	mTOR Rabbit Monoclonal Antibody	Beyotime, China	AF1648	1：1000
p-TOR (Ser2448)	mTOR (phospho-S2448) polyclonal antibody	Bioworld, China	BS4706	1：1000
lipin 1	Rabbit polyclonal to Lipin 1	Abcam, England	ab70138	1：1000
SREBP	SREBF1 Rabbit Polyclonal Antibody	Beyotime, China	AF8055	1：1000
CDKA	CDk1 Rabbit Polyclonal Antibody	Beyotime, China	AF0111	1：1000
H3	Histone H3 Rabbit Polyclonal Antibody	Beyotime, China	AF7101	1：1000
IgG	Goat Anti-Rabbit IgG	Thermo, USA	31460	1：10000

Table S4. Illumina sequencing statistics.

Sample	Number of raw reads	Number of clean reads	Number of bases after cleaning up (Gb)
Con_0h-1	95 271 544	94 548 592	13.92
Con_0h-2	101 911 968	101 013 786	14.84
Con_0h-3	113 215 080	112 620 330	16.69
Exp_6h-1	106 492 622	105 983 858	15.71
Exp_6h-2	112 499 404	111 987 420	16.57
Exp_6h-3	86 047 616	85 622 264	12.66
Exp_12h-1	101 547 914	101 072 388	14.92
Exp_12h-2	108 105 028	107 577 102	15.91
Exp_12h-3	106 415 586	106 010 310	15.60
Exp_24h-1	99 552 780	99 145 176	14.72
Exp_24h-2	116 643 390	116 045 394	17.23
Exp_24h-3	100 491 998	100 007 890	14.80
Exp_48h-1	96 508 286	96 137 410	14.26
Exp_48h-2	94 102 724	93 775 532	13.83
Exp_48h-3	86 776 154	86 446 370	12.83
Exp_60h-1	90 796 986	90 532 522	13.43
Exp_60h-2	90 162 082	89 888 030	13.33
Exp_60h-3	99 393 072	98 982 188	14.62

Table S5. Assembly quality statistics.

Gene number	GC percentage (%)	N50 number	N50 length (bp)	Max length (bp)	Min length (bp)	Average length (bp)	Total assembled bases
74,360	64.30	13 935	2 169	48 005	201	1 537	114 307 106

Table S6. Overview of all unigene annotation.

Annotation database	Number of Unigenes	Percentage (%)
Annotated in Nr	52 108	70.08
Annotated in Swissprot	30 530	41.06
Annotated in KOG	22 464	30.21
Annotated in GO	13 772	18.52
Annotated in KO	30 982	41.66
Annotated in Pfam	40 543	54.52
Annotated in SMART	13 440	18.07
Annotated in all databases	3 343	4.50
Annotated in at least one database	56 512	76.00
Total Unigenes	74 360	100.00

Table S7. Assignment of the identified metabolites to lipid classes and amount of lipid species identified in this study.

Lipid type	Description	Class	# of species identified
Cer	Ceramide	Sphingolipids (SLs)	13
CL	Cardiolipin	Phospholipid (PLs)	2
CmE	Campesterol ester	Sterol Lipids (STLs)	1
DG	Diacylglycerol	Glycolipids (GLs)	21
FA	Fatty acid	Fatty acids (FAs)	13
Hex1Cer	Hexose ceramide	Sphingolipids (SLs)	7
MGDG	Monogalactosil-diacylglicerol	Glycolipids (GLs)	27
OAHFA	(O-acyl)-1-hydroxy fatty acid	Fatty acids (FAs)	5
PA	Phosphatidic acid	Phospholipid (PLs)	2
PC	Phosphatidylcholine	Phospholipid (PLs)	26
PE	Phosphatidylethanolamine	Phospholipid (PLs)	2
PG	Phosphatidylglycerol	Phospholipid (PLs)	14
PI	Phosphatidylinositol	Phospholipid (PLs)	5
PS	Phosphatidylserine	Phospholipid (PLs)	4
SM	Sphingomyelin	Sphingolipids (SLs)	1
SPH	Sphingosine	Sphingolipids (SLs)	5
TG	Triacylglycerol	Glycolipids (GLs)	96
WE	Wax ester	Fatty esters (FEs)	2

Table S8. Annotation and accession numbers of Unigenes verified by qPCR.

Unigene ID	Annotation	Abbreviation	Accession numbers in GenBank database
Unigene0008422	acetyl-CoA carboxylase	*ACC*	XM_005788150.1
Unigene0023174	3-oxoacyl-[acyl-carrier-protein] synthase II	*FabF*	XM_005780026.1
Unigene0022129	3-oxoacyl-[acyl-carrier protein] reductase	*FabG*	XM_005787795.1
Unigene0023630	3-hydroxyacyl-[acyl-carrier-protein] dehydratase	*FabZ*	XM_005772130.1
Unigene0030522	enoyl-[acyl-carrier protein] reductase I	*FabI*	XM_005789274.1
Unigene0022848	carnitine O-acyltransferase 2	*CAT2*	XM_005784708.1
Unigene0003649	acetyl CoA oxidase	*ACOX*	XM_005768017.1
Unigene0044191	long-chain specific acyl-CoA dehydrogenase	*ACADS*	XM_005762654.1
Unigene0033009	acetyl-CoA acetyltransferase	*ACAT*	XM_005780889.1
Unigene0027907	glycerol kinase	*glpK*	XM_005768843.1
Unigene0039897	glycerol-3-phosphate dehydrogenase (NAD+)	*GPD*	XM_005768703.1
Unigene0002753	glycerol-3-phosphate O-acyltransferase	*GPAT*	XM_005774060.1
Unigene0032196	phosphatidate phosphatase	*LPP*	XM_005769462.1
Unigene0038589	triacylglycerol lipase	*TGL*	XM_005760724.1
Unigene0038983	acylglycerol lipase	*MGLL*	XM_005779767.1
Unigene0005883	serine palmitoyltransferase	*hSPT*	XM_005761941.1
Unigene0048914	3-dehydrosphinganine reductase	*KSR*	XM_005762413.1
Unigene0001578	sphingoid base N-stearoyltransferase	*CerS*	XM_005762024.1
Unigene0059254	dehydroceramide desaturase	*DCD*	XM_005765423.1
Unigene0064996	ceramide glucosyltransferase	*UGCG*	XM_005770209.1
Unigene0059709	glucosylceramidase	*GBA*	XM_005784801.1
Unigene0057891	sphingosine kinase	*SPK*	XM_005790888.1
Unigene0042369	phosphatidylinositol-3-kinase	*PI3K*	XM_005780980.1
Unigene0023672	Protein kinase B	*AKT*	XM_005761357.1
Unigene0022428	Putative target of rapamycin protein	*TOR*	XM_005781806.1
-	viral serine palmitoyltransferase	*vSPT*	CAZ69386.1
-	viral sphingoid base N-stearoyltransferase	*vCerS*	CAZ69354.1
-	viral dehydroceramide desaturase	*vDCD*	CAZ69746.1

-	viral fatty acid hydroxylase	*vFAH*	CAZ69370.1
-	viral phosphatidate phosphatase	*vLPP*	CAZ69415.1
-	viral triacylglycerol lipase	*vTGL*	CAZ69367.1

Table S9. Differentially expressed miRNAs in terms of log2 (Fold change) from the transcriptome and -ΔΔCt from qPCR.

miRNA	Comparative group	qPCR (-ΔΔCt)	Log2 Fold change
ehx-miR1-5p	Exp6h vs Con0h	1.09 ± 0.02	1.77
	Exp24h vs Con0h	1.33 ± 0.02	1.45
	Exp60h vs Con0h	-0.73 ± 0.02	-1.66
	Exp12h vs Exp6h	-0.57 ± 0.05	-1.46
	Exp24h vs Exp12h	0.81 ± 0.09	1.13
	Exp48h vs Exp24h	-0.60 ± 0.01	-1.23
	Exp60h vs Exp48h	-1.46 ± 0.02	-1.87
ehx-miR2-3p	Exp6h vs Con0h	-1.07 ± 0.03	-16.62
	Exp24h vs Exp12h	1.01 ± 0.04	2.21
ehx-miR3-3p	Exp24h vs Exp12h	0.28 ± 0.04	15.22
ehx-miR4-5p	Exp6h vs Con0h	1.72 ± 0.10	16.49
	Exp24h vs Con0h	0.51 ± 0.03	17.05
	Exp60h vs Con0h	0.66 ± 0.04	17.63
	Exp12h vs Exp6h	-2.24 ± 0.02	-16.49
	Exp24h vs Exp12h	1.03 ± 0.06	17.05
ehx-miR5	Exp24h vs Con0h	1.41 ± 0.02	1.92
	Exp48h vs Con0h	1.30 ± 0.05	2.49
	Exp60h vs Con0h	0.91 ± 0.03	2.32
	Exp24h vs Exp12h	1.50 ± 0.11	2.06
ehx-miR6-3p	Exp6h vs Con0h	0.32 ± 0.03	15.46
ehx-miR7-5p	Exp6h vs Con0h	0.73 ± 0.02	1.75
	Exp12h vs Exp6h	-1.08 ± 0.01	-1.37
ehx-miR8-3p	Exp24h vs Exp12h	1.19 ± 0.03	0.95
ehx-miR9-5p	Exp24h vs Exp12h	-4.14 ± 0.04	-15.04
ehx-miR10-3p	Exp24h vs Exp12h	0.85 ± 0.05	15.43
ehx-miR11-3p	Exp24h vs Exp12h	1.13 ± 0.08	16.25
	Exp24h vs Con0h	1.61 ± 0.03	16.25
ehx-miR12-5p	Exp60 vs Con0h	-0.56 ± 0.13	-1.15
ehx-miR13-3p	Exp60h vs Exp48h	-3.16 ± 0.05	-16.33
ehx-miR14-3p	Exp6h vs Con0h	0.22 ± 0.03	3.04
	Exp48h vs Exp24h	-0.26 ± 0.04	-15.36
ehx-miR15-3p	Exp12h vs Exp6h	-1.20 ± 0.02	-1.79
	Exp24h vs Exp12h	1.19 ± 0.04	1.18
ehx-miR16-3p	Exp6h vs Con0h	-0.67 ± 0.01	-15.68
ehx-miR17-5p	Exp24h vs Exp12h	1.29 ± 0.01	15.26
ehx-miR18-5p	Exp60h vs Exp48h	-3.01 ± 0.03	-16.01
ehx-miR19-5p	Exp6h vs Con0h	-1.05 ± 0.10	-1.03
	Exp12h vs Con0h	-2.36 ± 0.09	-3.80
	Exp60h vs Con0h	-1.93 ± 0.12	-16.73
	Exp12h vs Exp6h	-1.31 ± 0.01	-2.78

ehx-miR20-5p	Exp24h vs Con0h	-1.67 ± 0.06	-4.05
	Exp48h vs Con0h	-1.13 ± 0.10	-2.47
	Exp60h vs Con0h	-1.11 ± 0.09	-1.61
	Exp24h vs Exp12h	-0.81 ± 0.10	-3.83
	Exp48h vs Exp24h	0.54 ± 0.04	1.58
ehv-miR1-3p	Exp48h vs Exp24h	28.08 ± 0.13	25.84
	Exp60h vs Exp48h	0.01 ± 0.09	-0.14
ehv-miR2-3p	Exp48h vs Exp24h	28.28 ± 0.06	27.2
	Exp60h vs Exp48h	1.69 ± 0.02	1.78
ehv-miR3-3p	Exp48h vs Exp24h	24.24 ± 0.12	27.36
	Exp60h vs Exp48h	0.26 ± 0.04	-0.28
ehv-miR4-3p	Exp48h vs Exp24h	30.12 ± 0.08	28.02
	Exp60h vs Exp48h	-3.35 ± 0.03	-2.04
ehv-miR5-3p	Exp48h vs Exp24h	25.21 ± 0.09	27.73
	Exp60h vs Exp48h	-1.03 ± 0.03	-1.45
ehv-miR6-5p	Exp60h vs Exp48h	1.32 ± 0.00	23.34
ehv-miR7-5p	Exp48h vs Exp24h	28.07 ± 0.24	26.82
	Exp60h vs Exp48h	-0.42 ± 0.14	-2.46

Table S10. Plant-type targets of host miRNAs which were related to lipid metabolism.

Unigene ID	Annotation	miRNAs	Pathways
Unigene0023630	beta-hydroxyacyl-acp dehydratase precursor	ehx-miR5	Fatty acid biosynthesis
Unigene0040727	3-oxoacyl	ehx-miR19-5p	Fatty acid biosynthesis
Unigene0059968	acyl-CoA synthetase, long-chain	ehx-miR15-3p	Fatty acid biosynthesis
Unigene0049330	aldehyde dehydrogenase (NAD+)	ehx-miR14-3p	Fatty acid degradation
Unigene0026672	alpha-galactosidase	ehx-miR16-3p	Sphingolipid metabolism
Unigene0050579	lecithin:cholesterol acyltransferase	ehx-miR16-3p	Glycerophospholipid metabolism
Unigene0063980	DAO-domain-containing protein	ehx-miR15-3p	Glycerophospholipid metabolism
Unigene0003767	probable phospholipid hydroperoxide glutathione peroxidase	ehx-miR15-3p	Arachidonic acid metabolism

Supplementary Figures

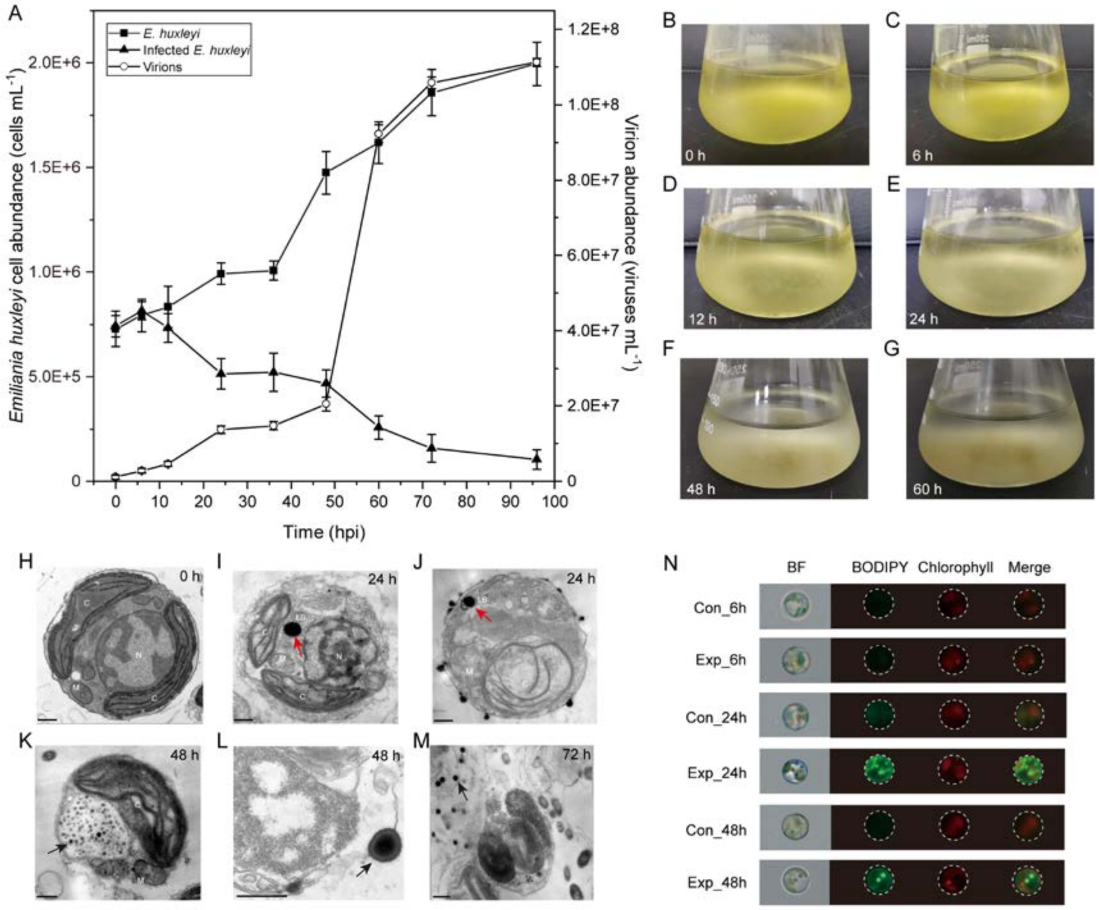

Figure S1. Infection dynamics, ultrastructure, and neutral lipid accumulation during EhV infection. (**A**) Abundance of algae in the uninfected control cultures, and algae and virus in the infected cultures measured by flow cytometry (mean ± SD, n = 3). (**B**) to (**G**) *E. huxleyi* BOF92 cultures of non-infected cells (**B**) and cells at 6 hpi (**C**), 12 hpi (**D**), 24 hpi (**E**), 48 hpi (**F**), and 60 hpi (**G**). (**H**) to (**M**) Transmission electron micrographs of control cells (H) and cells infected by virus at 24 hpi (**I**), (**J**), 48 hpi (**K**), (**L**), and 72 hpi (**M**) (scale bar in (**L**): 200 nm; others: 500 nm). (**G**) Fluorescence microscopy images of control cells or cells infected with the lytic virus at 6 hpi, 24 hpi and 48 hpi. Images are composites of bright field (BF), BODIPY fluorescence (green), chlorophyll autofluorescence (red) and a merged image.

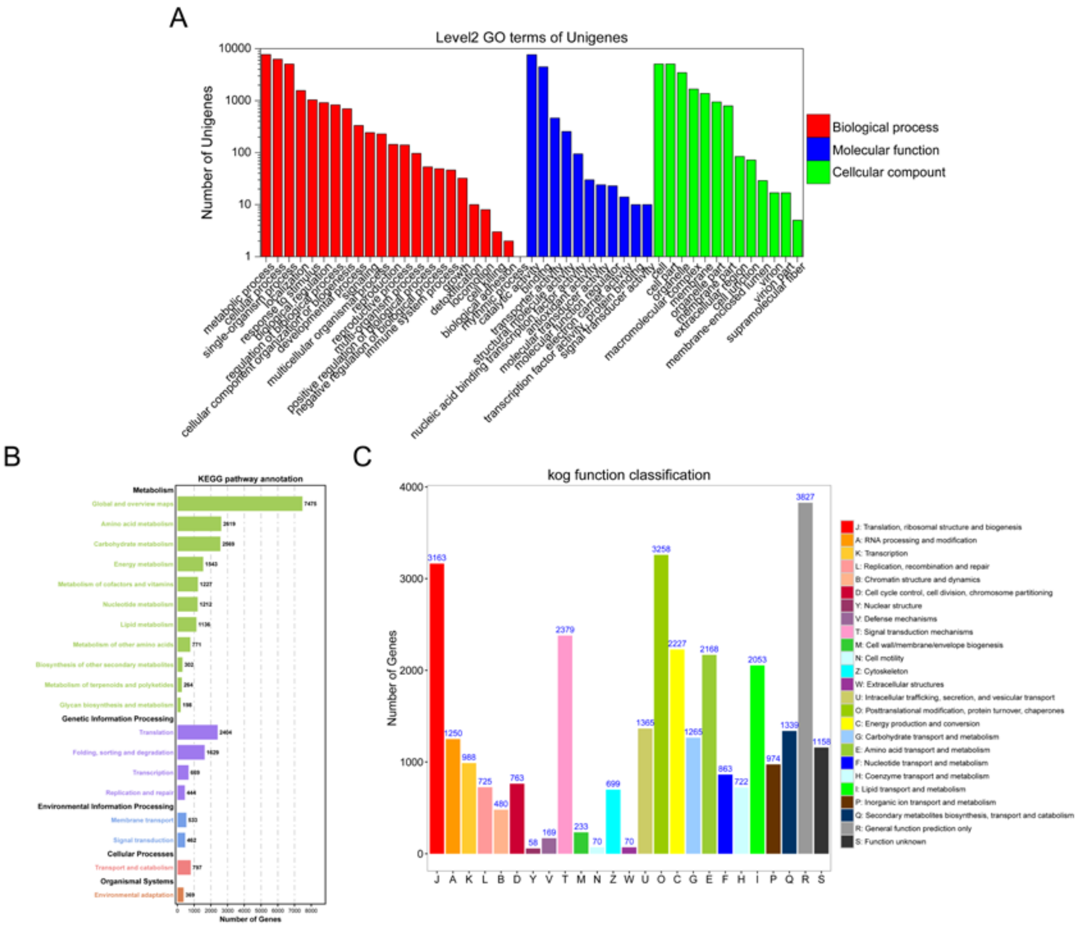

Figure S2. Gene annotation of the *de novo E. huxleyi* transcripts. (**A**) Gene Ontology analysis of unigenes; (**B**) KEGG pathway annotation; (**C**) KOG-based functional classification.

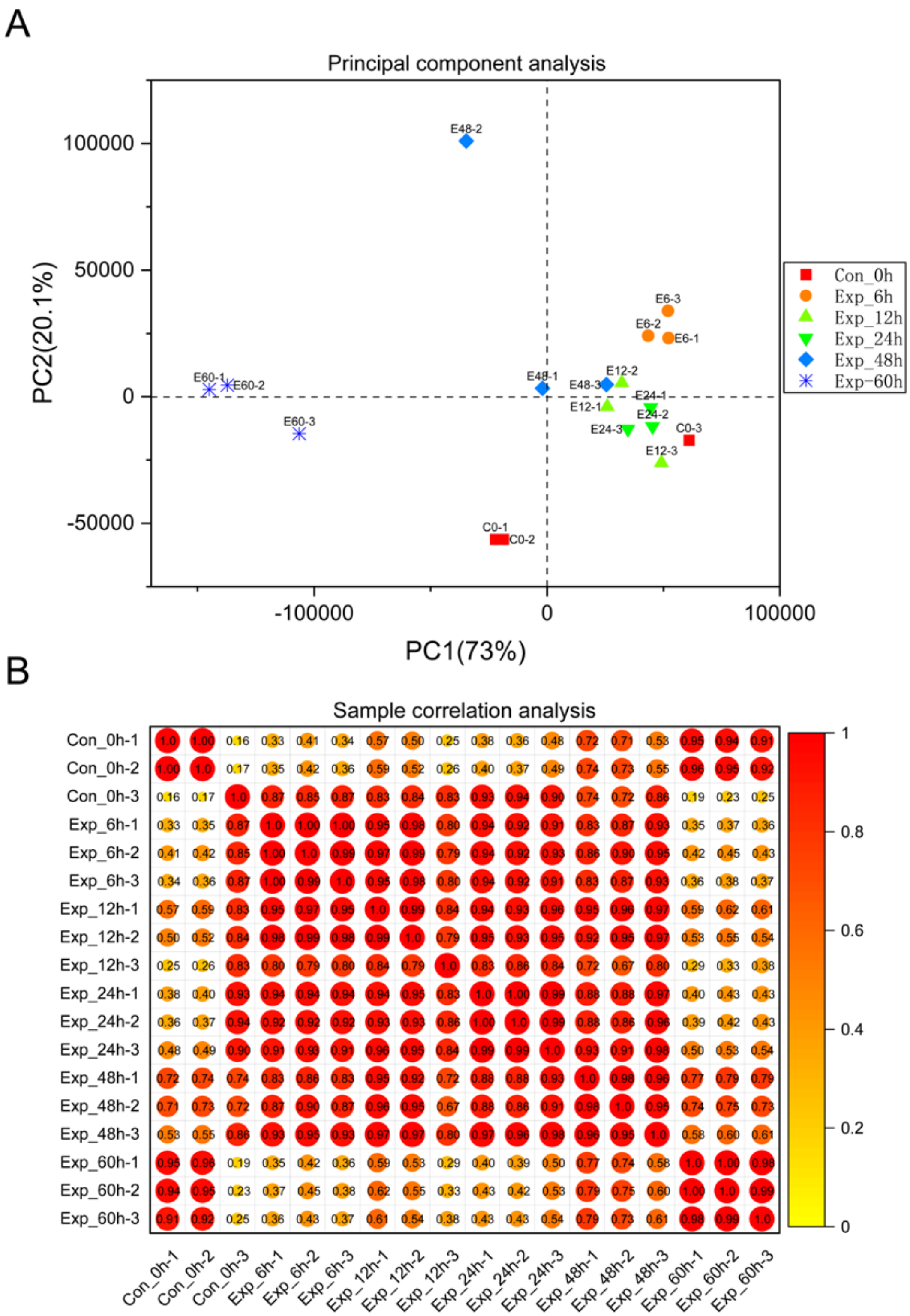

Figure S3. Relationship analysis of samples. (**A**) Principal component analysis (PCA) for reads per kilobase per million mapped reads (RPKM); (**B**) Correlation analysis of replicas (n=3).

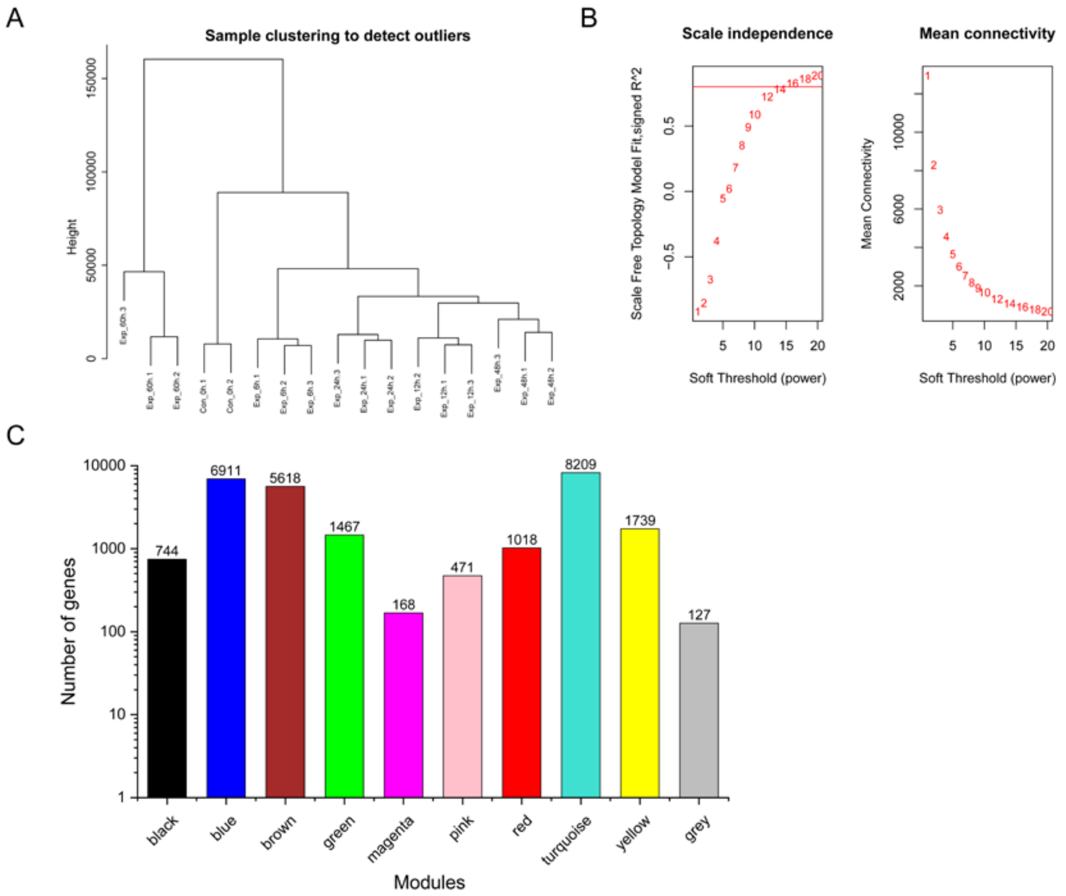

Figure S4. Weighted gene co-expression network analysis (WGCNA). (**A**) Sample clustering to detect outliers. All samples except Con_0h-3 were included to perform WGCNA. (**B**) Scale independence and mean connectivity by calculating 26472 genes. β was selected as 16. (**C**) The number of genes in each module.

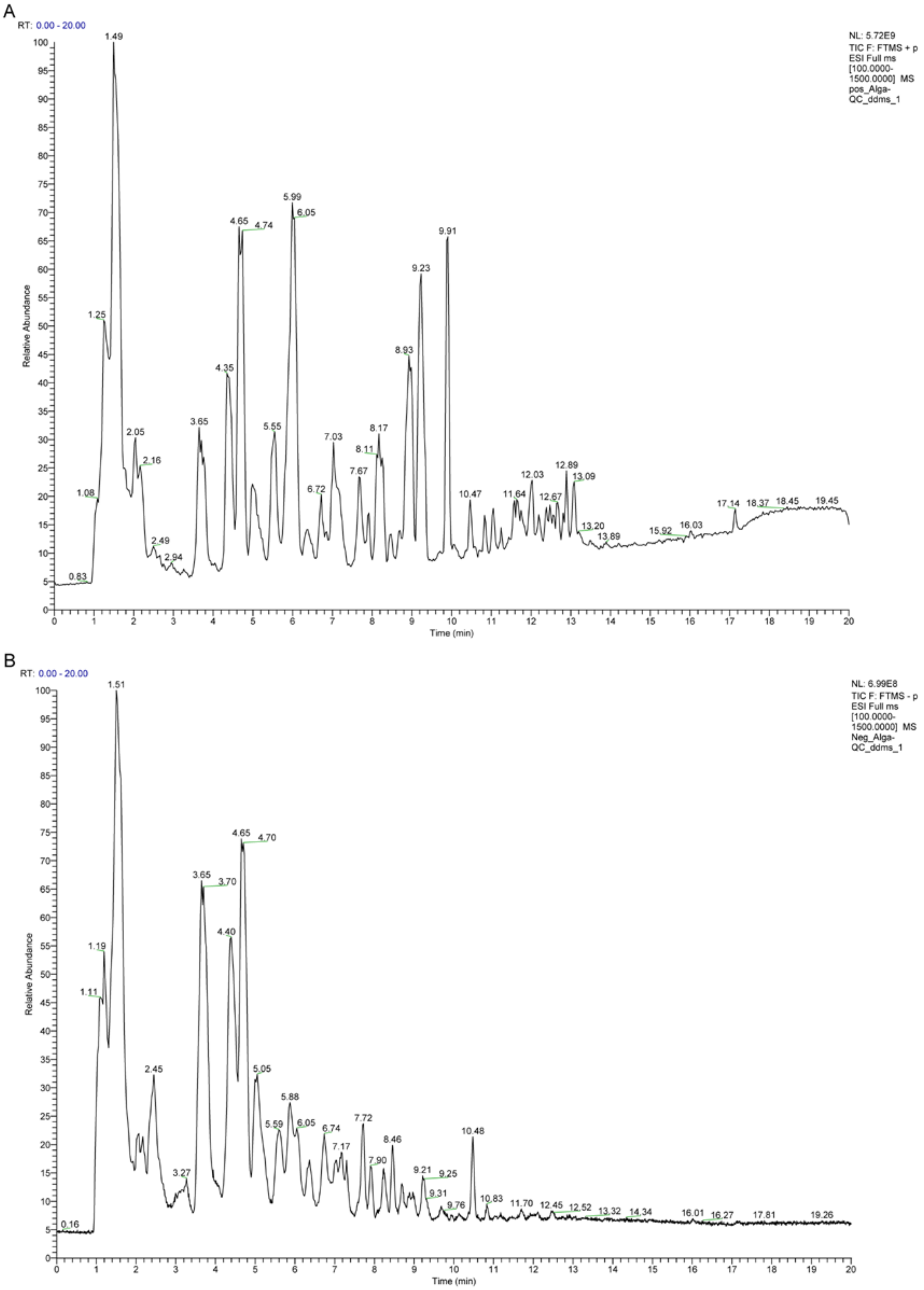

Figure S5. Base peak chromatography of total lipid profile. Pooled cell extract was acquired using LC-MS in ESI positive (**A**) and negative mode (**B**), respectively. The number of lipid species identified for each lipid class (described in Supplemental Dataset 1) is specified (#).

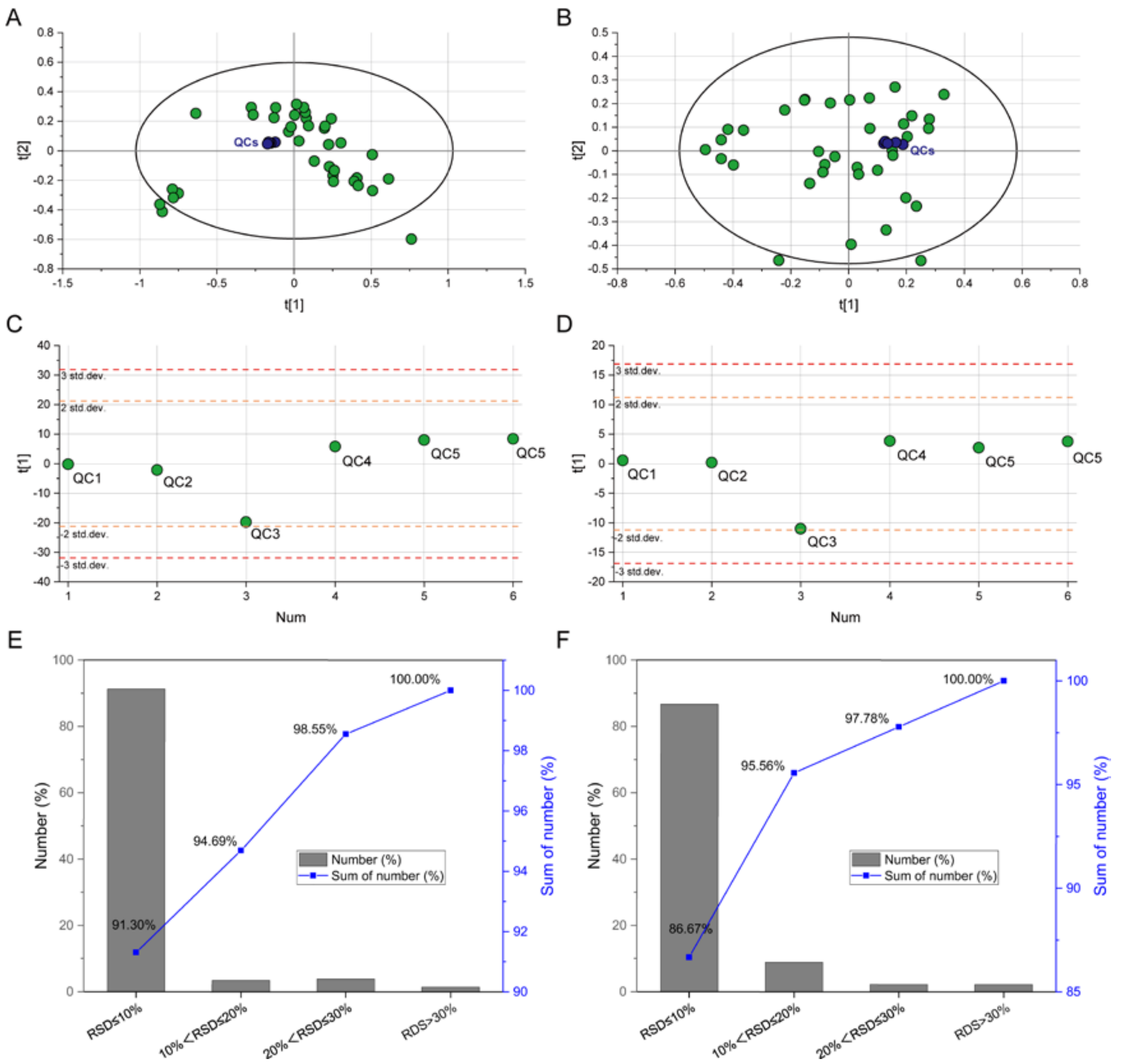

Figure S6. Quality evaluation of total lipid profile. (**A**) and (**B**) are score plots for all samples from positive and negative analysis mode, respectively. (**C**) and (**D**) are PCA score plots for QC samples from positive and negative analysis mode, respectively. (**E**) and (**F**) indicate the distributions of %RSD for lipid species among all QC samples from positive and negative mode, respectively.

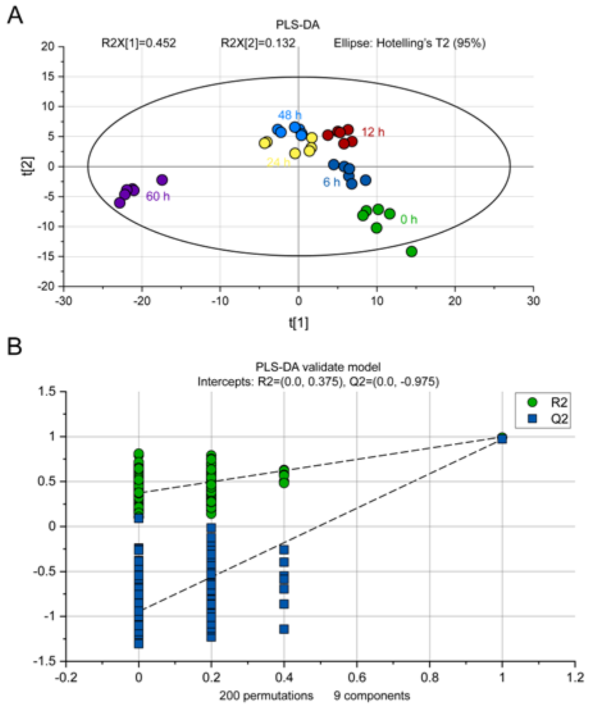

Figure S7. PLS-DA model of lipidomic samples. (A) Score plot of PLS-DA model. (B) PLS-DA validate model. Based on response permutation test with 200 iterations, the PLS-DA model were validated without overfitting.

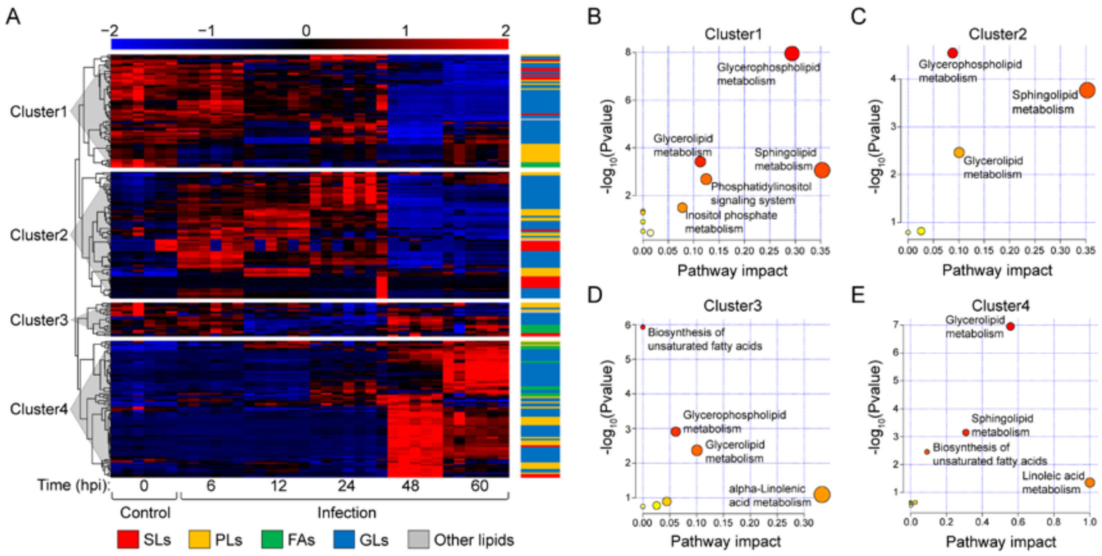

Figure S8. Global lipidomic profiles and pathway enrichment analysis during the large-scale infection process. (**A**) Global lipid profiles during infection. The alterations in the contents of 246 lipid species in infected and control cells were visualized by clustergram representation. The assignment of each detected lipid with four major lipid classes was distinguished by different colors. SLs, sphingolipids; PLs, phospholipids; FAs, fatty acids; GLs, glycerolipids. (**B**) to (**E**) Overviews of the KEGG pathway analysis based on metabolite clusters as displayed in (**A**).

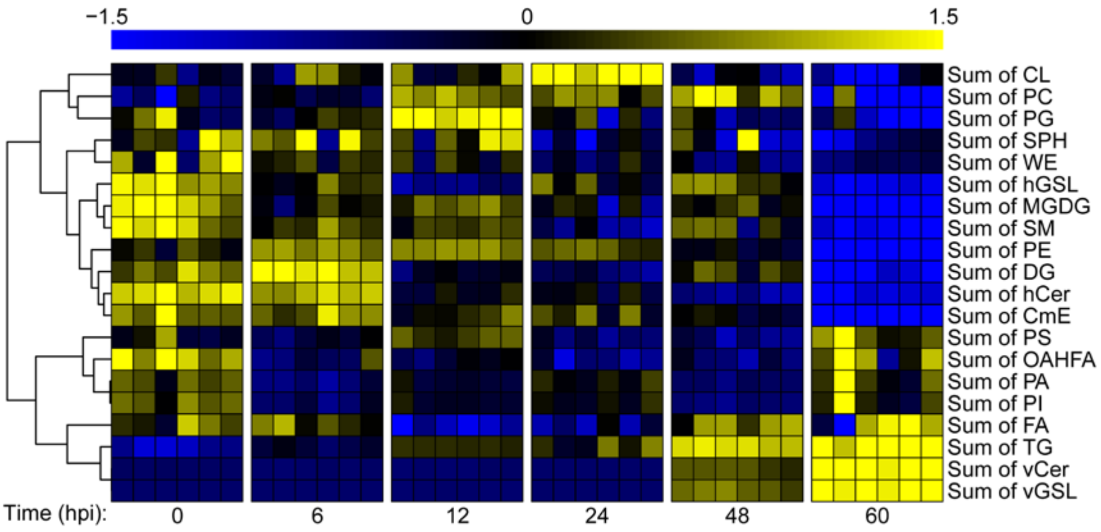

Figure S9. Heatmap visualization of total lipid profile. The sum of responses from each lipid class was normalized using z-score and subjected to hierarchical clustering to visualize the variation tendencies across different groups

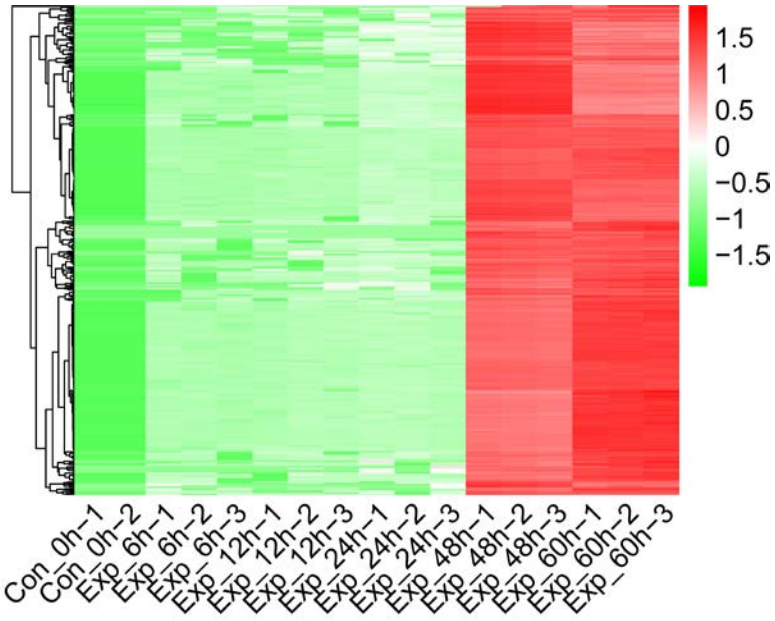

Figure S10. The hierarchical heatmap of all 443 viral genes. Red color indicates up regulation and green color indicates down regulation. Every column represents a sample and every row represents a mRNA probe.

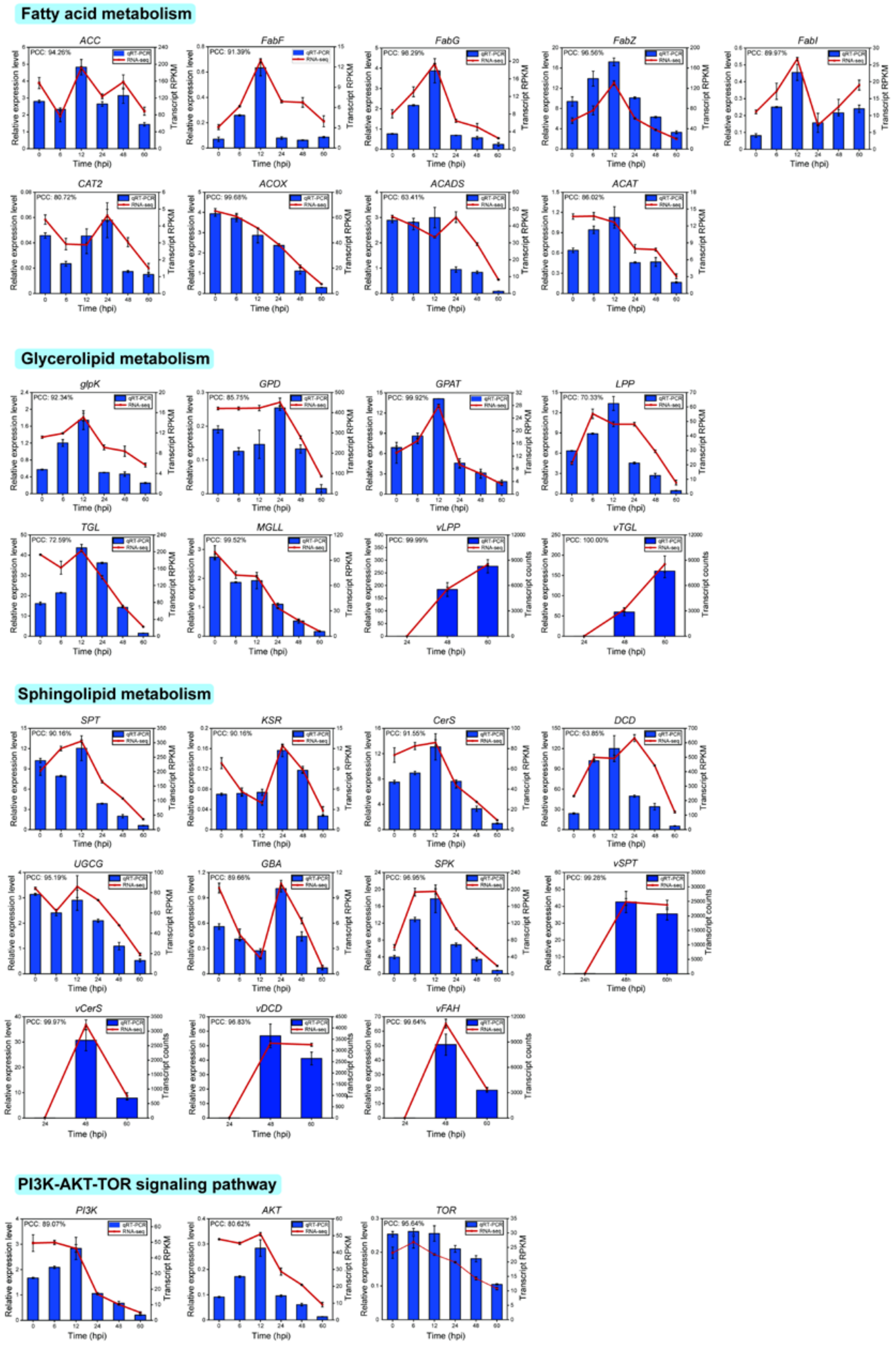

Figure S11. The qRT-PCR results of selected genes relevant to lipid metabolism. In each plot, red line means the expression trend of RNA-seq results, blue bars represent the relative quantification by qPCR. Pearson correlation coefficient (PCC) was calculated and shown on the top left of each plot.

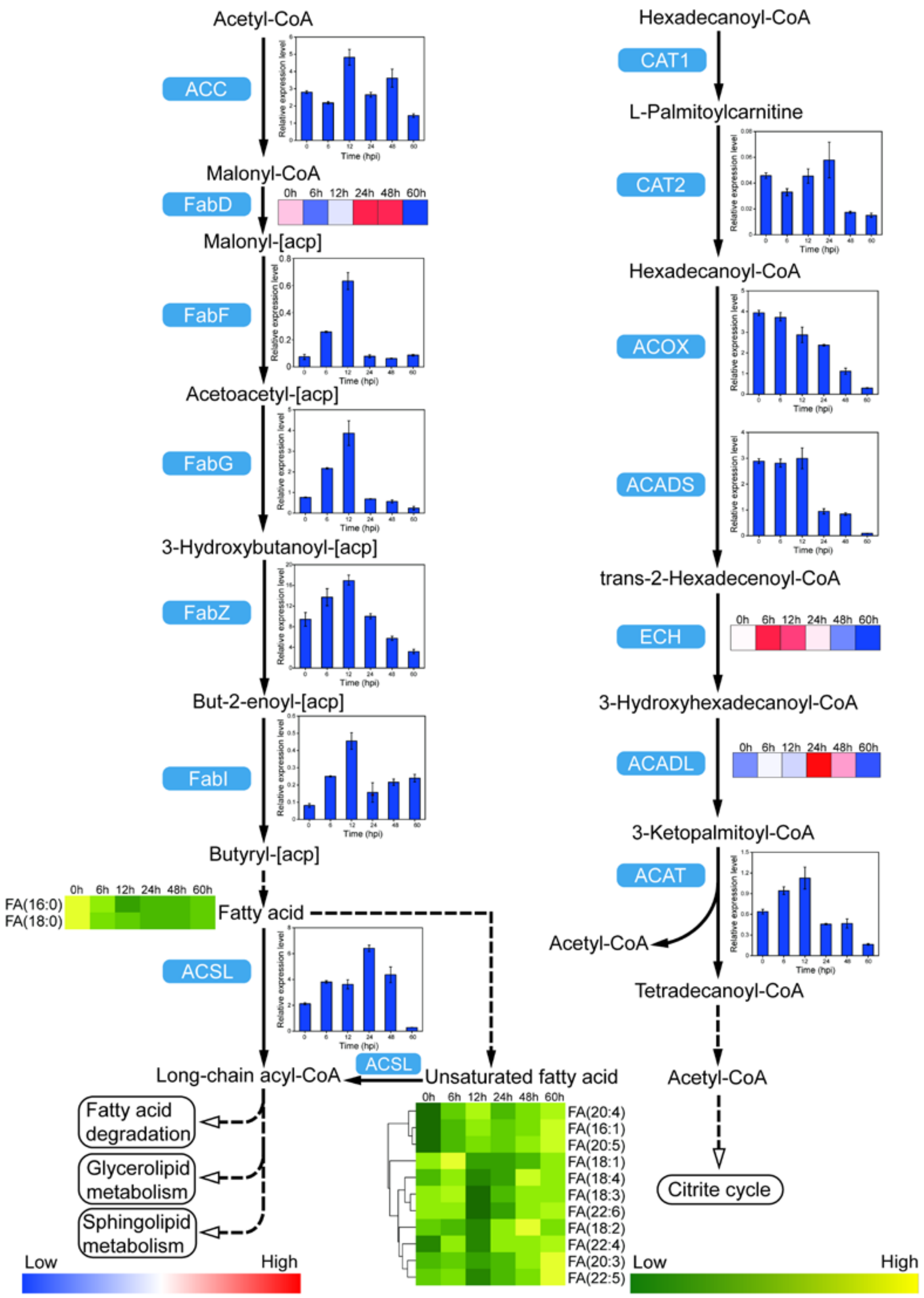

Figure S12. Integrated metabolic map of fatty acid biosynthesis and β-oxidation. Inserted heatmaps presented normalized intensities of genes (mean, n=3, blue-red heatmap) and lipids (mean, n=6, green-yellow heatmap) in noninfected *E. huxleyi* cells (0 h) and cells infected by the lytic virus at 6, 12, 24, 48 and 60 hpi. blue, low gene expression; red, high gene expression; green, low metabolite abundance; yellow, high metabolite abundance. Dashed lines with solid arrows refer to abbreviated processes. Dashed lines with hollow arrows refer to connections via known metabolic pathways. For abbreviations of genes, see Supplemental Dataset 2.

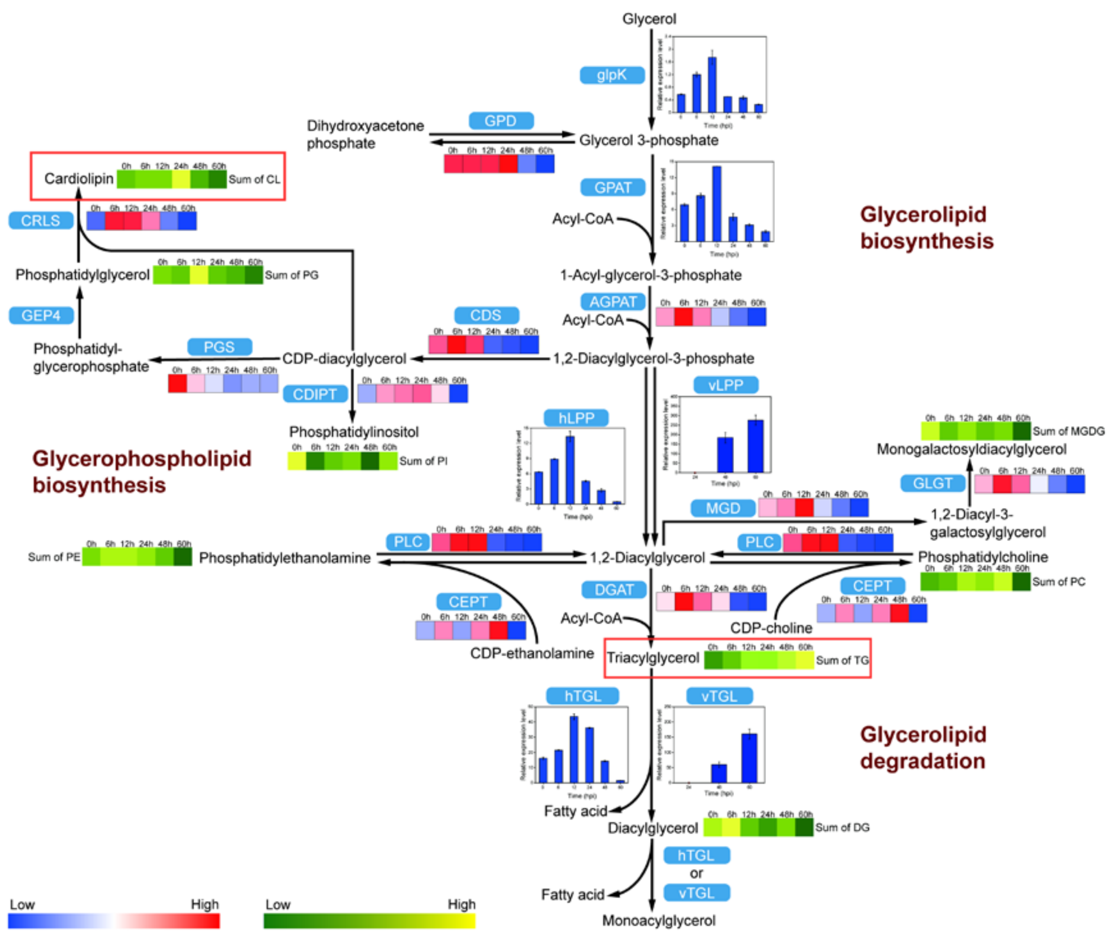

Figure S13. Integrated metabolic map of glycerolipid metabolism and glycerophospholipid biosynthesis. Inserted heatmaps presented normalized intensities of genes (mean, n=3, blue-red heatmap) and lipids (mean, n=6, green-yellow heatmap) in noninfected *E. huxleyi* cells (0 h) and cells infected by the lytic virus at 6, 12, 24, 48 and 60 hpi. blue, low gene expression; red, high gene expression; green, low metabolite abundance; yellow, high metabolite abundance. The levels of CLs and TGs are noted by red rectangle. For abbreviations of genes, see Supplemental Dataset 2.

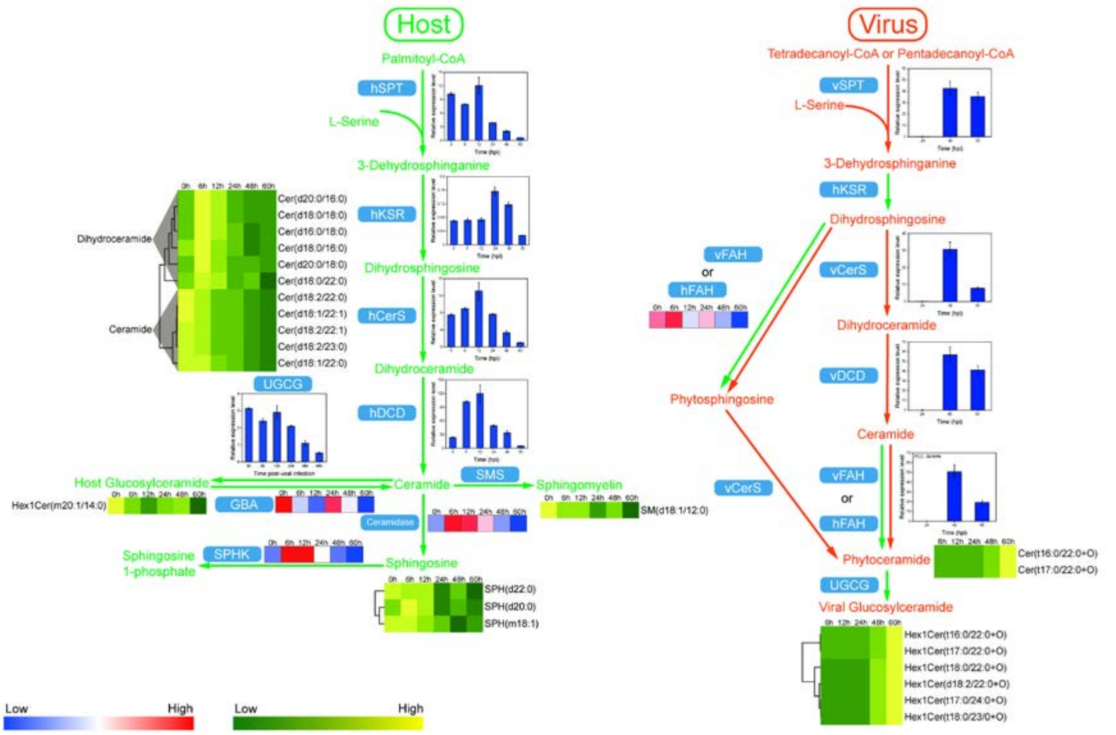

Figure S14. Integrated metabolic map of host and viral sphingolipid metabolism. Host and viral metabolic processes were differentiated by light green and orange color, respectively. Inserted heatmaps presented normalized intensities of genes (mean, n=3, blue-red heatmap) and lipids (mean, n=6, green-yellow heatmap) in noninfected *E. huxleyi* cells (0 h) and cells infected by the lytic virus at 6, 12, 24, 48 and 60 hpi. blue, low gene expression; red, high gene expression; green, low metabolite abundance; yellow, high metabolite abundance. For abbreviations of genes, see Supplemental Dataset 2.

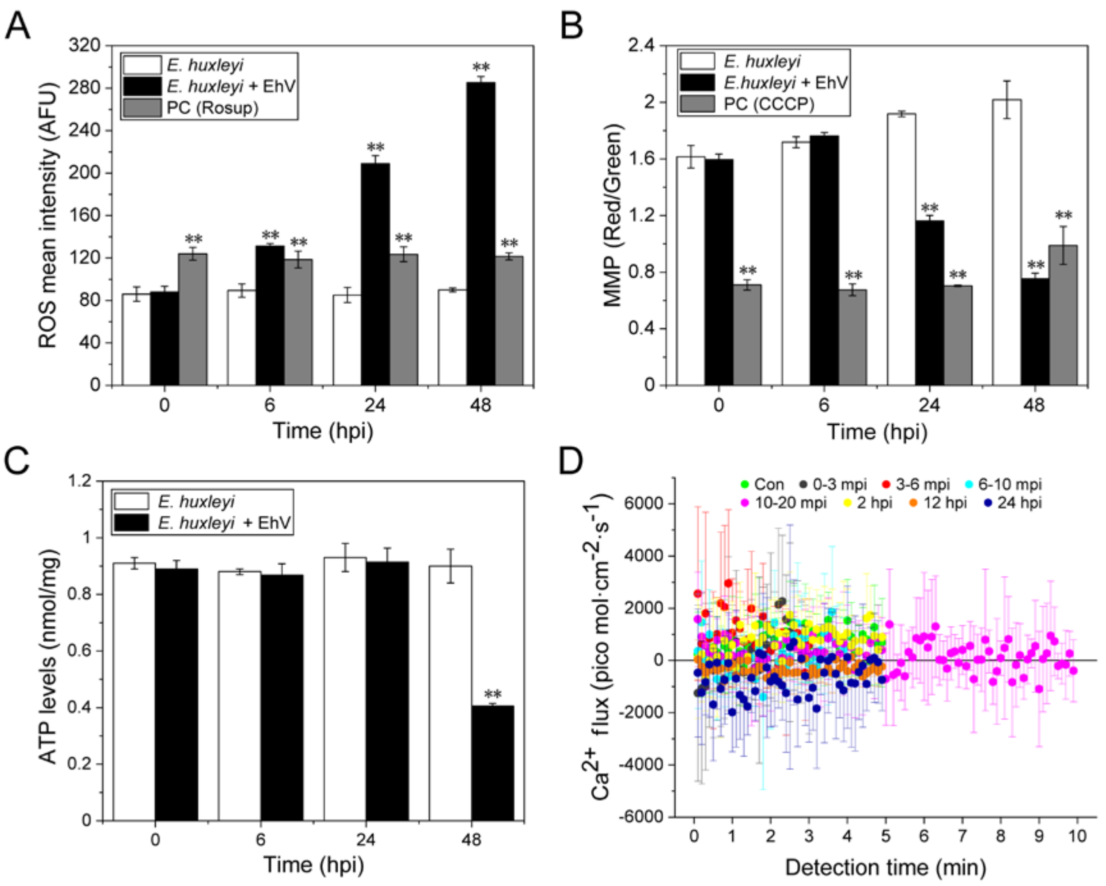

Figure S15. Detection of mitochondrial functions and Ca^{2+} influx during EhV infection. (**A**) Cellular ROS measurements. PC, positive control. Rosup is a kind of compound mixture that can induce intracellular ROS production. (**B**) Mitochondrial membrane potential (MMP) measurements. CCCP, carbonyl cyanide 3-chlorophenylhydrazone. (**C**) Mitochondrial ATP measurements. The experiment results above were presented as mean ± SD, n = 3. (**$p < 0.01$). (**D**) Measurements of Ca^{2+} flux through cell membrane. Ca^{2+} flux was firstly detected during 20 minutes after virus transient treatment. Then, Ca^{2+} flux was detected for 5 minutes at 2, 12 and 24 hpi, respectively. The results were presented as mean ± SD, n = 12.

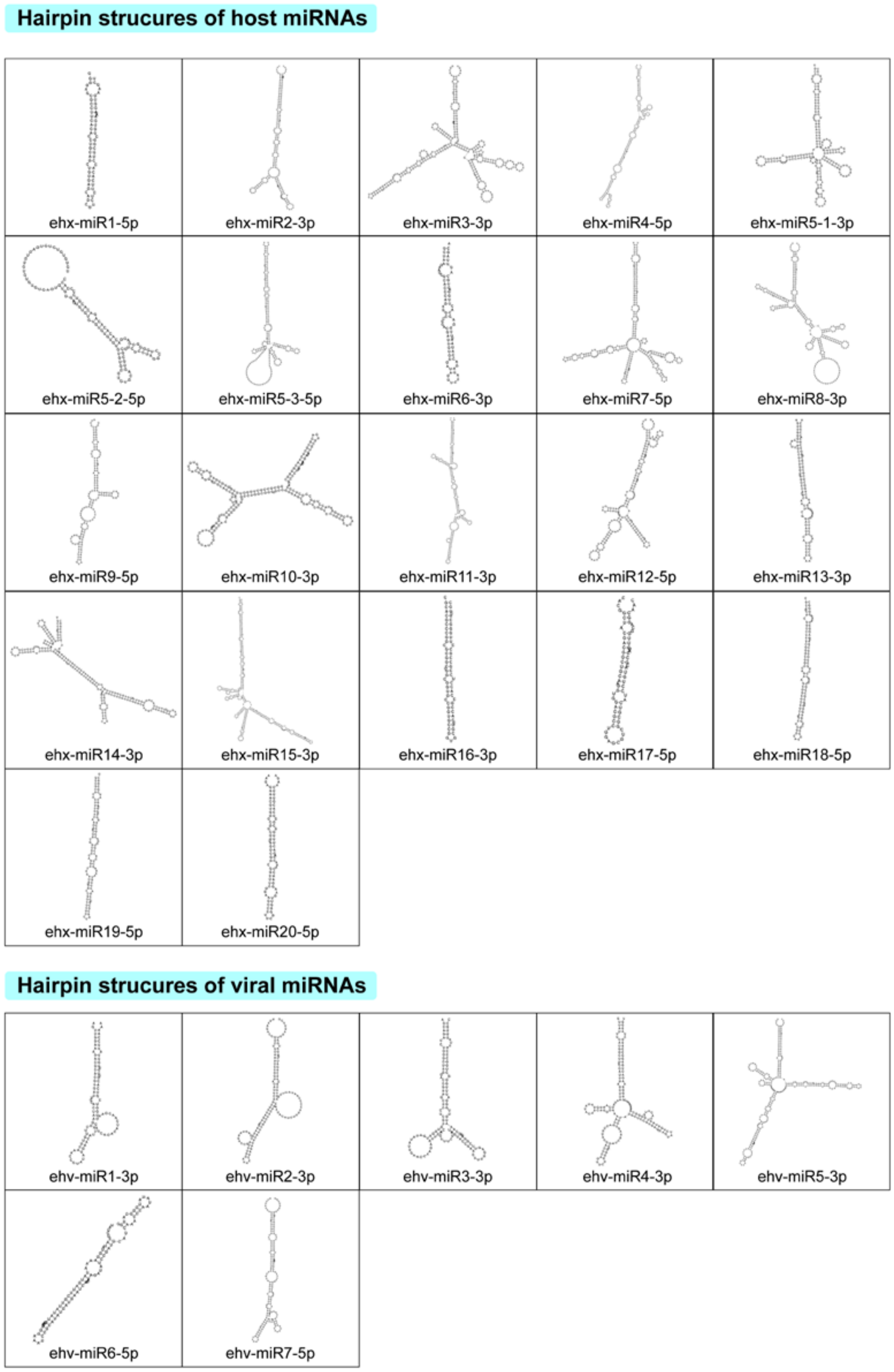

Figure S16. The hairpin structures of DE host and viral miRNAs. These structures were predicted by RNA structure, an online software. More details of miRNAs were shown in Supplemental Dataset 4.

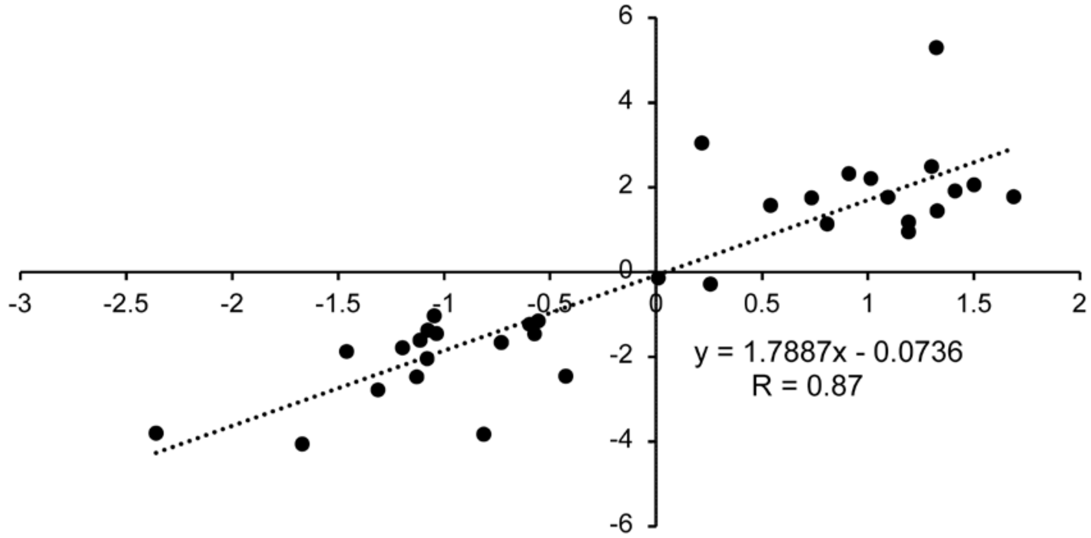

Figure S17. The regression analysis of gene expression between the results of qRT-PCR and miRNA-seq. X-axis represents the qRT-PCR results; Y-axis represents the miRNA-seq results.

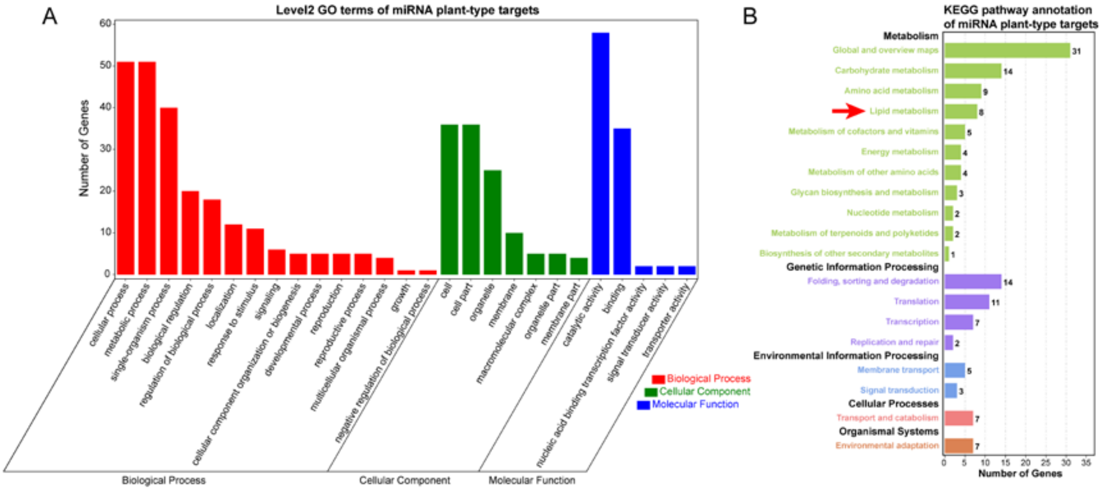

Figure S18. Gene annotation of the plant-type targets of host miRNAs. (**A**) Gene Ontology annotation; (**B**) KEGG pathway annotation. Lipid metabolism annotation in KEGG is noted by red arrow and the details are shown in Table S10.

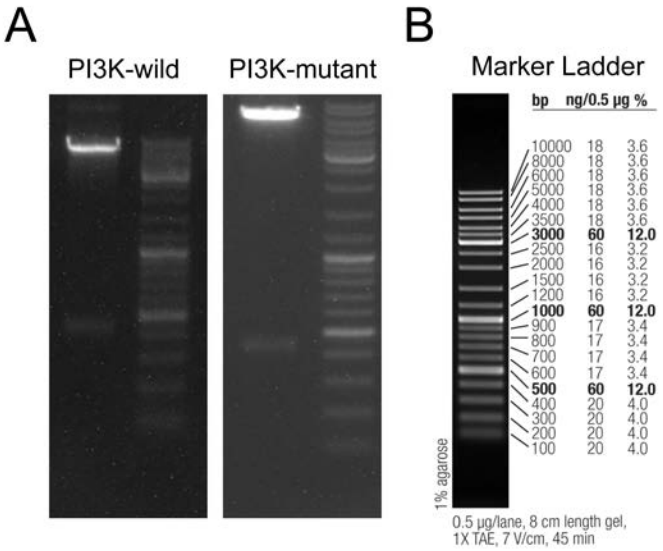

Figure S19. **The agarose gel electrophoresis results of restriction enzyme digestion of each recombinant psi-CHECK2 vectors.** (**A**) The lengths of digested sequences were all around 400 bp. (**B**) The maker ladder used in agarose gel electrophoresis assay.
